# Circulating Cell‐Free DNAs as a Biomarker and Therapeutic Target for Acetaminophen‐Induced Liver Injury

**DOI:** 10.1002/advs.202206789

**Published:** 2023-04-10

**Authors:** Madi Sun, Peiyu Chen, Kai Xiao, Xiang Zhu, Zhibin Zhao, Chenyang Guo, Xuan He, Tongfei Shi, Qingguo Zhong, Yong Jia, Yu Tao, Mingqiang Li, Kam W. Leong, Dan Shao

**Affiliations:** ^1^ School of Biomedical Sciences and Engineering South China University of Technology Guangzhou International Campus Guangzhou Guangdong 510630 China; ^2^ National Engineering Research Center for Tissue Restoration and Reconstruction South China University of Technology Guangzhou International Campus Guangzhou Guangdong 510630 China; ^3^ School of Medicine South China University of Technology Guangzhou International Campus Guangzhou Guangdong 510006 China; ^4^ Laboratory of Biomaterials and Translational Medicine The Third Affiliated Hospital Sun Yat‐sen University Guangzhou Guangdong 510006 China; ^5^ School of Nursing Jilin University Changchun Jilin 130021 China; ^6^ Department of Systems Biology Columbia University New York NY 10032 USA; ^7^ Guangdong Provincial Key Laboratory of Biomedical Engineering Key Laboratory of Biomedical Materials and Engineering of the Ministry of Education South China University of Technology Guangzhou Guangdong 510006 China

**Keywords:** acetaminophen, acute liver injuries, biomarkers, cell‐free DNA, inflammation, oxidative damage

## Abstract

Acetaminophen (APAP) overdose is a leading cause of drug‐induced liver injury and acute liver failure, while the detection, prognosis prediction, and therapy for APAP‐induced liver injury (AILI) remain improved. Here, it is determined that the temporal pattern of circulating cell‐free DNA (cfDNA) is strongly associated with damage and inflammation parameters in AILI. CfDNA is comparable to alanine aminotransferase (ALT) in predicting mortality and outperformed ALT when combined with ALT in AILI. The depletion of cfDNA or neutrophils alleviates liver damage, while the addition of cfDNA or adoptive transfer of neutrophils exacerbates the damage. The combination of DNase I and *N*‐acetylcysteine attenuates AILI significantly. This study establishes that cfDNA is a mechanistic biomarker to predict mortality in AILI mice. The combination of scavenging cfDNA and reducing oxidative damage provides a promising treatment for AILI.

## Graphical abstract

This study establishes that circulating cell‐free DNA (cfDNA) can be used as a biomarker to predict mortality of acetaminophen‐induced liver injury (AILI). Circulating cfDNA is a causative factor that amplifies liver damage and inflammation through neutrophils. The critical factors of inflammation and oxidative damage in driving AILI can be ameliorated by the combination of DNase I and *N*‐acetylcysteine.

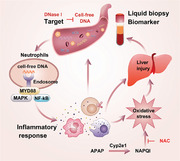

## 1 Introduction

Acetaminophen (APAP)‐induced liver injury (AILI) is one of the most common causes of death due to acute liver failure in the developed countries and is considered a significant public health problem in developing world.^[^ ^1^ ^]^ Although *N*‐acetylcysteine (NAC) is effective in treating AILI at the early phase of intoxication, its efficacy is markedly diminished in patients who present themselves late after APAP overdose.^[^ ^2^ ^]^ Unfortunately, at that point, liver transplantation may be the only definitive life‐saving procedure.^[^ ^1b^ ^]^ A dependable biomarker that can predict the prognosis of AILI would be valuable in deciding the best course of treatment.^[^ ^3^ ^]^ Among existing biomarkers, alanine aminotransferase (ALT) is generally used in clinical practice to predict hepatotoxicity. Nevertheless, there is a clear unmet need for biomarkers that can reflect the various aspects of APAP hepatotoxicity, along with biomarkers specific to predict its progression or resolution.^[^ ^4^ ^]^ Exploring the mechanistic biomarkers and therapeutic targets is highly required for the precision‐guided AILI management.

Damage‐associated molecular patterns (DAMPs), including histone, high mobility group box 1 (HMGB1), and mitochondria DNA, provide a bridge between damage and inflammation, both of which play crucial roles in the pathophysiology of AILI.^[^ ^5^ ^]^ In such a scenario, DAMPs released from APAP‐damaged hepatocytes activate immune cells via pattern recognition receptors, then drive cytokine and chemokine‐based cascades for the initiation of inflammation.^[^ ^6^ ^]^ As a consequence, circulating immune cells are recruited to boost the systemic inflammatory responses.^[^ ^6^, ^7^ ^]^ Targeting the connection between damage and inflammation may provide fruitful insights to benefit the prediction and treatment of AILI. Cell‐free DNA (cfDNA), including mitochondrial DNA (mtDNA), nuclear DNA, and neutrophil extracellular traps (NETs), activates toll‐like receptor 9 (TLR9)‐mediated pro‐inflammatory signaling of immune cells and contributes to the magnitude and duration of inflammation.^[^ ^5^, ^8^ ^]^ cfDNA may also be recognized as a promising biomarker in various inflammatory liver diseases.^[^ ^9^ ^]^ Circulating cfDNA has been demonstrated to be elevated in APAP overdose patients.^[^ ^10^ ^]^ Moreover, the degradation of cfDNA by DNase I alleviates APAP hepatotoxicity in animal models.^[^ ^11^ ^]^ Nevertheless, little is known about how cfDNA links the damage and inflammation during the time course of APAP hepatotoxicity. Notably, the potential of cfDNA has not been adequately evaluated in the prediction and therapy of AILI.

In the current study, we aimed to explore the temporal pattern of circulating cfDNA in the interaction between liver damage and inflammation and confirm its potential to predict the outcome and prognosis of AILI. We sought to investigate the mechanisms of cfDNA in the setting of AILI, and further explored the combination of DNase I and NAC as a potential therapy for AILI.

## 2 Results

### 2.1 cfDNA Is a Viable Biomarker to Predict the Outcome and Prognosis of AILI

**Figure 1:**
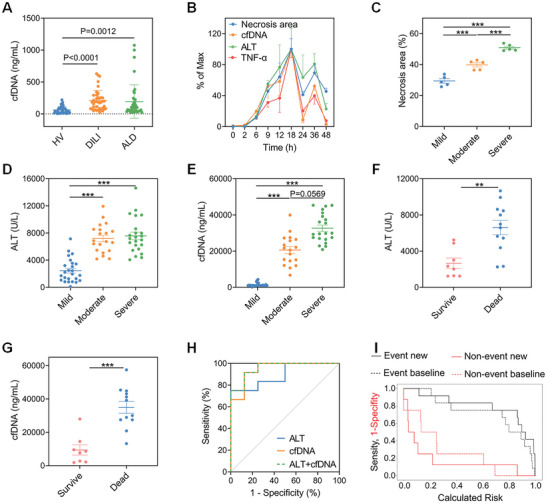
cfDNA is a viable biomarker to predict the outcome and prognosis of AILI. A) Circulating cfDNA in HV (*n* = 40), DILI patients (*n* = 40), and ALD patients (*n* = 40). B) The levels of liver necrosis area, circulating cfDNA, ALT, and TNF‐*α* (*n* = 3–5 for each time point) in moderate AILI. C–E) The levels of necrosis area (*n* = 5 for each group), circulating ALT, and cfDNA (*n* = 18–25 for each group) at 12 h in mild, moderate, and severe AILI model. F,G) The levels of circulating ALT and cfDNA were measured at 12 h in dead or survival mice of severe AILI (550 mg kg^−1^, *n* = 20). H) ROC curve analysis showed the potential of cfDNA, ALT, and ALT + cfDNA to predict the outcome of AILI (550 mg kg^−1^, *n* = 20). I) Risk assessment of baseline model (ALT) and new model with the addition of cfDNA (ALT + cfDNA) to predict outcome. Subjects who did not survive (event, black line) and survive (non‐event, red line) are shown respectively (*n* = 20). Data are expressed as means ± SD, ^*^ *p* < 0.05, ^**^ *p* < 0.01, ^***^ *p* < 0.001.

We first examined circulating cfDNA levels in healthy volunteers (HV) (*n* = 40), drug‐induced liver injury (DILI) patients (*n* = 40), and alcoholic liver disease (ALD) patients (*n* = 40) as described in Table S1, Supporting Information. Significantly higher cfDNA levels in DILI patients (*p* < 0.0001) and ALD patients (*p* = 0.0012) versus healthy volunteers were observed (**Figure** **1**A). To characterize the temporal pattern of damage and inflammation in AILI, we quantified a panel of factors through multi‐timepoint analysis (0, 2, 6, 9, 12, 18, 24, 36, and 48 h after APAP) in a moderate AILI model (Figure S1A, Supporting Information). Obvious necrosis in liver histology was observed at 6 h (Figure S1B,C, Supporting Information), while ALT and aspartate aminotransferase (AST) levels markedly increased at 6 h after APAP challenge (Figure S1D,E, Supporting Information). Histopathology analysis and elevated serum transaminase levels together established peak liver damage at 18 h after APAP challenge. Similarly, the level of circulating cfDNA increased at 2 h and peaked at 18 h after APAP challenge (Figure S1F, Supporting Information). Meanwhile, the temporal pattern of serum tumor necrosis factor‐*α* (TNF‐*α*) and HMGB1 also revealed a similar trend (Figure S1G,H, Supporting Information), indicating the potential association among cfDNA, liver damage, and inflammation in a progressive and time‐dependent manner. We then concluded the correlation between cfDNA and markers of liver damage and inflammation in Figure 1B. We found that the increase of circulating cfDNA was significantly correlated with elevated necrosis area (Spearman *r* = 0.8237 [95% CI: 0.5697–0.9340, *p* < 0.0001]), ALT (Spearman *r* = 0.9267 [95% CI: 0.8528–0.9642, *p* < 0.0001]), AST (Spearman *r* = 0.8935 [95% CI: 0.7897–0.9476, *p* < 0.0001]), and TNF‐*α* (Spearman *r* = 0.9300 [95% CI: 0.8420–0.9698, *p* < 0.0001]) (Figure S2A–H, Supporting Information). A significant relationship was also observed between liver damage, inflammation factors, and elevated HMGB1, a classic DAMP during AILI (Figure S2I–P, Supporting Information). Nevertheless, the correlation of AILI markers with HMGB1 was weaker than that with cfDNA. We also compared the relationship between ALT and necrotic area (Spearman *r* = 0.7030 [95% CI: 0.4239–0.8602, *p* < 0.0001]), indicating a weaker correlation compared to cfDNA (Figure S2Q, Supporting Information). These findings confirmed that the temporal pattern of circulating cfDNA was highly associated with the liver damage and inflammation manner in APAP‐challenged mice.

On the basis of the temporal pattern of circulating cfDNA, we explored the predictive potential of cfDNA in AILI. We developed AILI model with different severity by varying the dosage of APAP, and the degree of damage was confirmed by determining the necrosis area (Figure 1C and Figure S3, Supporting Information). AILI mice with severe grade had the highest circulating cfDNA levels. The ALT levels of the moderate and severe models were both higher than the mild model, although with no significant difference between them (Figure 1D,E). Interestingly, cfDNA distinguished the degree of liver damage better than ALT, indicating its advantage in the characterization of AILI. We further explored the ability of cfDNA as a prognostic biomarker in a severe AILI model in the presence or absence of NAC. Both circulating cfDNA and ALT levels at 12 h after APAP challenge were significantly higher in non‐survived mice compared to survived mice (Figure 1F,G). cfDNA exhibited comparable ability for death prediction compared with ALT (AUROC 0.9479 vs 0.8958), and the cut‐off values were 17.66 µg mL^−1^ and 5340 U L^−1^ for cfDNA and ALT, respectively (Figure 1H and Table S2, Supporting Information). Although the specificity of cfDNA was inferior to ALT, its sensitivity was superior. Moreover, the addition of cfDNA improved the ability of ALT to predict death (AUROC 0.9583 vs 0.8958). Notably, integrated discrimination improvement (IDI) was 0.2158 (95%CI: 0.0426–0.3889), and net reclassification index (NRI) was 1.0833 (95%CI: 0.38–1.7866), further revealing the added value of cfDNA to ALT when predicting the prognosis of APAP challenge (Figure 1I and Table S3, Supporting Information). We concluded from these results that circulating cfDNA was a viable indicator for AILI, which provided a better prognosis prediction when cfDNA was added to the basal model of ALT.

### 2.2 cfDNA Amplifies Liver Damage and Inflammation Partly through Neutrophils

**Figure 2:**
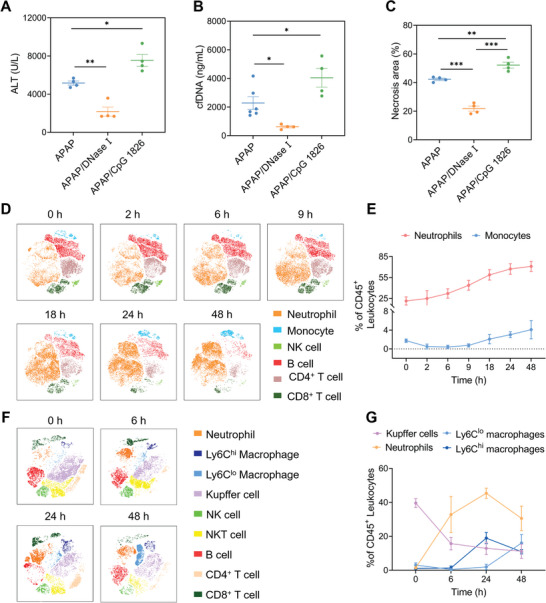
The causative role of cfDNA and variation of leukocytes during AILI. Mice were treated with the DNase I at 3/10 h after APAP challenge (450 mg kg^−1^). CpG ODN 1826 was administrated at 3 h after APAP. A–C) Circulating ALT, cfDNA, and necrosis areas were determined at 24 h. D) t‐SNE analysis of leukocytes in peripheral blood at different time points after APAP. E) The dynamics of neutrophils and monocytes in peripheral blood. F) t‐SNE analysis of leukocytes in liver at different time points after APAP. G) The dynamics of Kupffer cells, neutrophils, Ly6C^lo^, and Ly6C^hi^ macrophages. *n* = 3–6 for each group. Data are expressed as means ± SD, ^*^ *p* < 0.05, ^**^ *p* < 0.01, ^***^ *p* < 0.001.

Having demonstrated the potential of cfDNA as a biomarker, we sought to investigate the role of cfDNA in the instigation of AILI. First, when we treated APAP‐challenged mice with DNase I to degrade cfDNA, liver damage was alleviated remarkedly by reduced ALT and necrosis area (**Figure** **2**A–C and Figure S4A, Supporting Information). In contrast, the addition of CpG ODN 1826 significantly aggravated liver damage. DNase I or CpG ODN 1826 treatment could not induce obvious liver damage in normal mice (Figure S4B, Supporting Information). Since circulating cfDNA drove inflammatory response in multiple diseases, we sought to decipher the relationship between cfDNA and hepatic leukocytes in AILI. We quantified the circulating immune cells at different time points (0, 2, 6, 9, 18, 24, and 48 h) after APAP challenge by flow cytometry (Figure S5A, Supporting Information). This overview was established by algorithm t‐distributed stochastic neighbor embedding (t‐SNE) analysis to represent the population clustering (Figure 2D). Circulating neutrophils responded quickly to insult and displayed a time‐dependent increase until 48 h after APAP challenge, while the percentage of circulating monocytes exhibited an initial decrease before a subsequent increase (Figure 2E). The NK cells, T cells, and B cells showed a continuous decline (Figure S5B, Supporting Information). Next, we quantified the liver immune cell and drew t‐SNE map of leukocytes in liver at 0, 6, 24, and 48 h after APAP challenge (Figure 2F and Figure S6, Supporting Information). After 6 h following AILI, there was a massive infiltration of neutrophils concomitant with a remarkable reduction of resident Kupffer cells (KCs) (Figure 2G). Importantly, the hepatic neutrophils continuously increased to a maximum proportion at 24 h before declining, whereas the KCs decreased until 48 h. Ly6C^hi^ CD11b^+^ macrophages with pro‐inflammatory phenotype infiltrated abundantly at 24 h but reduced at 48 h. Meanwhile, Ly6C^lo^ CD11b^+^ macrophages with pro‐resolving phenotype showed a slight decrease at 6 h followed by a massive infiltration up to 48 h.

**Figure 3:**
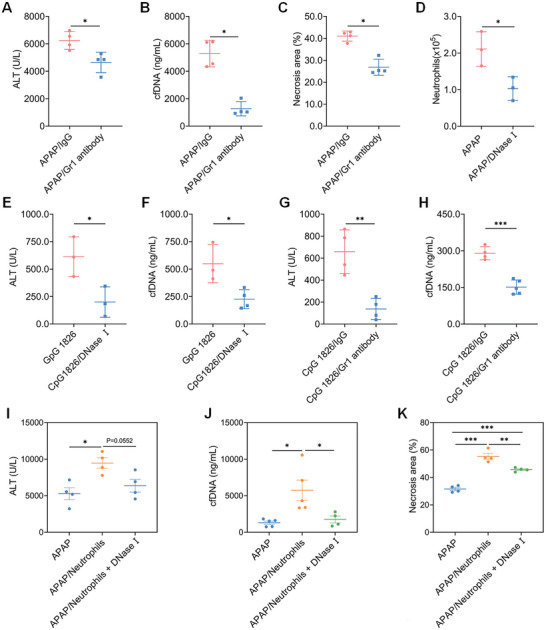
cfDNA amplifies liver damage and inflammation partly through neutrophils. Mice were treated with anti‐mouse Ly6G/Ly6C or IgG2b (100 µg/dose, i.p.) at 24 and 4 h prior to APAP (250 mg kg^−1^) or CpG 1826 (0.5 mg kg^−1^). A–C) Circulating ALT, cfDNA, and necrosis areas were determined at 24 h after APAP. D) Variation of hepatic neutrophils in AILI mice after DNase I treatment. Mice were treated with DNase I at 3/10 h after CpG 1826. E–H) Circulating ALT and cfDNA levels after DNase I treatment or after depletion of neutrophils at 24 h in CpG‐induced liver injury. *n* = 3–5 for each group. Bone marrow‐derived neutrophils were adoptively transferred into AILI mice (i.v.) and DNase I was administrated at 3 and 10 h after APAP. I–K) Circulating ALT, cfDNA, and necrosis areas were determined at 24 h after APAP. *n* = 3–5 for each group. Data are expressed as means ± SD, ^*^ *p* < 0.05, ^**^ *p* < 0.01, ^***^ *p* < 0.001.

Seeing that neutrophils and macrophages were recruited to the inflamed liver, we investigated whether those cells play crucial roles in the liver damage and inflammation of AILI. The depletion of neutrophils significantly decreased the circulating cfDNA levels and attenuated liver damage in a mild AILI model (**Figure** **3**A–C and Figure S7A,B, Supporting Information). Notably, greatly higher levels of cfDNA and exacerbated liver damage were found after macrophage depletion (Figure S7C–F, Supporting Information). As expected, degradation of cfDNA by DNase I remarkedly reduced the population of infiltrating neutrophils, but did not affect the number of total liver macrophages (Figure 3D and Figure S7G, Supporting Information). Consistent with previous results, the protective effects of cfDNA scavenging and neutrophils depletion were observed in CpG‐induced liver injury mice, while macrophage depletion increased live damage in the same model (Figure 3E–H and Figure S7H, Supporting Information). We further performed an adoptive transfer experiment to explore the relation between cfDNA and neutrophils during AILI. As shown in Figure 3I–K, adoptive transfer of neutrophils resulted in significantly elevated ALT levels and necrosis area, along with higher circulating cfDNA levels. However, the addition of DNase I suppressed the neutrophil‐exacerbated liver injury (Figure 3I–K and Figure S7I, Supporting Information). Furthermore, CpG‐activated neutrophils induced higher ALT and cfDNA levels in normal mice (Figure S7J,K, Supporting Information). Collectively, these findings supported the view that neutrophils were one of the important targets of circulating cfDNA during AILI. We believed that as a mechanistic biomarker and causative factor, cfDNA amplified liver damage and inflammation partly through neutrophils.

### 2.3 Combination of DNase I and NAC Protects Liver Damage in AILI

**Figure 4:**
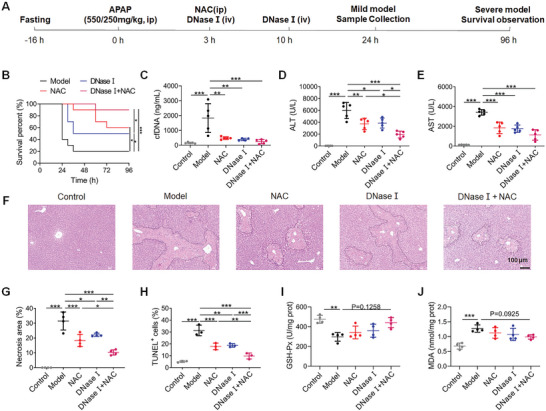
Combination of DNase I and NAC protects liver damage in AILI. A) Scheme of the experimental design. B) The survival rate after indicated treatment in the severe AILI model (550 mg kg^−1^, *n* = 10 for each group), ^*^ *p* < 0.05 and ^***^ *p* < 0.001 by Kaplan–Meier survival analysis. C–E) The levels of circulating cfDNA, ALT, and AST at 24 h after APAP (250 mg kg^−1^). F) Representative H&E staining of liver. Original magnification, ×100; Scale bar: 100 µm. G–J) The levels of necrosis area, TUNEL^+^ cells, GSH‐Px, and MDA in liver at 24 h after APAP (250 mg kg^−1^, *n* = 3–5 for each group). Data are expressed as means ± SD, ^*^ *p* < 0.05, ^**^ *p* < 0.01, ^***^ *p* < 0.001.

Having illustrated cfDNA was a mechanistic biomarker and causative factor in AILI, we investigated the potential of cfDNA as a therapeutic target by screening the dose of DNase I in a mild AILI model. Intravenous injection (i.v.) of 80 U/dose of DNase I attenuated hepatic damage, while lower and higher doses of DNase I did not show benefits (Figure S8A,B, Supporting Information). Although NAC is used widely in the clinic, we also demonstrated that early delivery of NAC by intraperitoneal injection (i.p.) provided strong hepatoprotective effects, but delayed administration (6 h) showed little protective efficacy (Figure S8C,D, Supporting Information). Based on these findings, we investigated whether the combination of DNase I and NAC would improve the AILI therapy. After optimizing the combination regimen, we established that the combination of DNase I (80 U/dose, i.v., 3 and 10 h) and NAC (150 mg kg^−1^, i.p., 3 h) exhibited the best hepatoprotective effect (**Figure** **4**A and Figure S8E,F, Supporting Information). Single administration of DNase I or NAC decreased the mortality from 80% in the control group to 50% or 40%, respectively, while combination of DNase I and NAC reduced it further to 10% (Figure 4B). In addition, we detected the cfDNA and ALT levels at 12 h to perform receiver‐operating characteristic (ROC) analysis and quantify the IDI and NRI metrics (Figure S9 and Tables S3 and S4, Supporting Information). Notably, either cfDNA or ALT alone was correlative with death after treatment. But the addition of cfDNA improved the prediction accuracy, which is consistent with the results presented above (Figure 1H,I).

In the mild AILI model, we found that either single or combination therapy significantly reduced the levels of circulating cfDNA, ALT, and AST, along with liver necrosis and apoptosis (Figure 4C–H and Figure S10A, Supporting Information). Correspondingly, the variation of oxidative stress‐related markers, including glutathione peroxidase (GSH‐Px) and malondialdehyde (MDA) were reversed partly after treatment (Figure 4I,J). The combination therapy of DNase I and NAC rendered less liver damage and oxidative stress than DNase I or NAC alone. Additionally, there was no significant change in the levels of circulating biochemical indicators or pathological analysis of the major organs after DNase I and NAC exposure in normal mice (Figure S11, Supporting Information). We also established a mild AILI model and NAC/DNase I were administrated at 10 h after APAP overdose. The results showed that delayed administration of NAC could not attenuate damage, while the DNase I delivery in the later phase still work to reverse the phenotypes partly, which further indicated the notion of cfDNA as a therapeutic target (Figure S10B–F, Supporting Information). Taken together, these findings suggested that cfDNA was not only a useful biomarker, but also a valuable therapeutic target in AILI. The combination of DNase I and NAC could provide stronger protective effects relative to monotherapies and without additional systematic toxicity at the optimal regimen.

### 2.4 Mechanism of DNase I Attenuating Inflammatory Response in AILI Model

**Figure 5:**
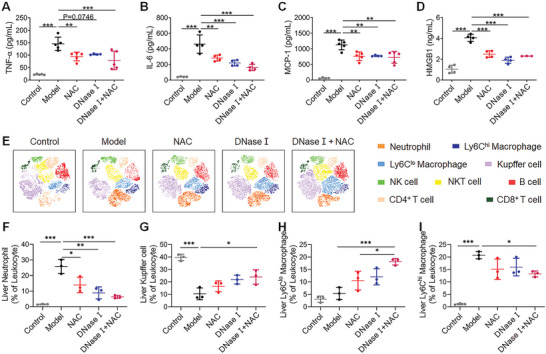
DNase I attenuates inflammatory response in AILI model. A–D) The levels of circulating TNF‐*α*, IL‐6, MCP‐1, and HMGB1 at 24 h after APAP (250 mg kg^−1^). Data are expressed as means ± SD (*n* = 4–5 for each group). E) The t‐SNE analysis of hepatic leukocytes at 24 h. F–I) Variation of hepatic neutrophils, Kupffer cells, Ly6C^lo^, and Ly6C^hi^ macrophages at 24 h following APAP (*n* = 3 for each group). Data are expressed as means ± SD, ^*^ *p* < 0.05, ^**^ *p* < 0.01, ^***^ *p* < 0.001.

Given the crucial role of cfDNA in inflammation, we next investigated how DNase I affected the inflammatory response in AILI. Administration of DNase I and/or NAC significantly reduced the elevation of circulating TNF‐*α*, interleukin‐6, monocyte chemoattractant protein‐1, and HMGB1 (**Figure** **5**A–D). Circulating immune cells were quantified at 24 h after APAP challenge by flow cytometry (Figure S12A, Supporting Information). Administration of DNase I significantly abrogated the elevated circulating neutrophils and monocytes (Figure S12B,C, Supporting Information). Phenotypic characterization of the liver leukocytes was carried out to further clarify the hepatic inflammation (Figure 5E and Figure S13, Supporting Information). After DNase I treatment, the percentage of hepatic neutrophils in AILI mice reduced from 25.77% to 8.85%, while the reduction of KCs was partly recovered (10.36% vs 21.83%) (Figure 5F,G and Figure S13A, Supporting Information). Moreover, preponderant phenotype of hepatic macrophages gradually shifted from pro‐inflammatory Ly6C^hi^ to pro‐resolving Ly6C^lo^ (Figure 5H,I and Figure S13A, Supporting Information). In agreement with these results, immunohistochemical staining (IHC) for Ly6G displayed a lower frequency of neutrophils in liver after treatment (Figure S14, Supporting Information). NAC also alleviated inflammation in AILI partially. However, the combination of DNase I and NAC displayed superior anti‐inflammatory effects relative to monotherapies.

### 2.5 Combination of DNase I and NAC Reverses Inflammation and Oxidative Stress in AILI

**Figure 6:**
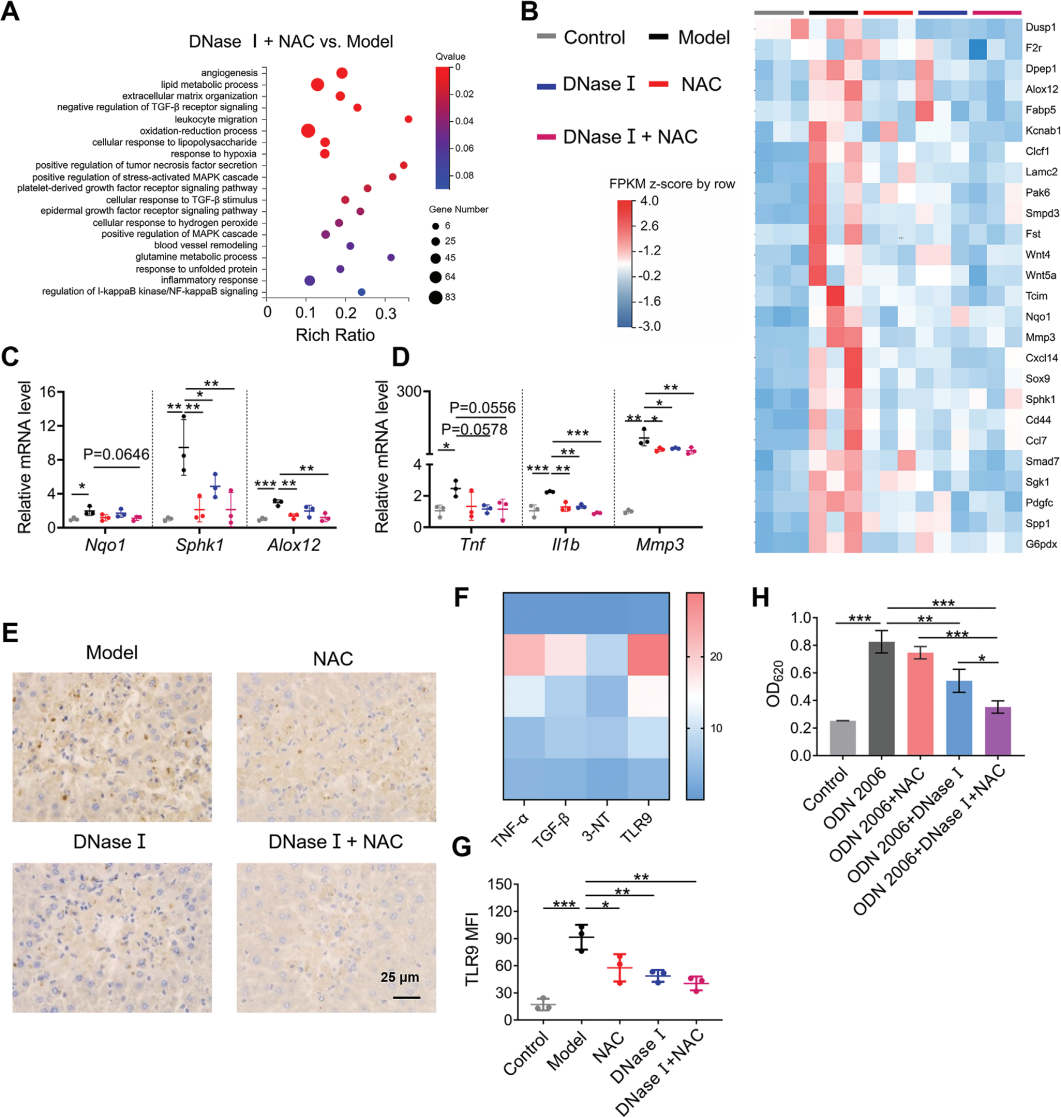
Combination of DNase I and NAC reverses inflammation and oxidative stress in AILI. A) The GO functional enrichment analysis of DEGs between DNase I + NAC and Model at 24 h following APAP (250 mg kg^−1^). B) Heat maps of significantly DEGs involved in oxidative stress and inflammation. C,D) qRT‐PCR of oxidative stress and inflammation related genes (*n* = 3 for each group). E) Heat maps of IHC H‐scores of TNF‐*α*, TGF‐*β*, 3‐NT, and TLR9. F) IHC staining of TLR9 at 24 h following APAP. Original magnification, ×400; Scale bar: 25 µm. G) The expression of TLR9 in neutrophils was determined by flow cytometry at 24 h following APAP. H) The activation of HEK‐Blue TLR9 reporter cells by CpG ODN 2006 was inhibited by DNase I and combination therapy. *n* = 3–4 for each group. Data are expressed as means ± SD, ^*^ *p* < 0.05, ^**^ *p* < 0.01, ^***^ *p* < 0.001.

To further elucidate the mechanisms of the combination treatment, we performed RNA sequencing of the liver at 24 h after APAP challenge. The differentially expressed genes (DEGs) between different comparison groups were displayed via VENN figure (Figure S15A, Supporting Information) and Volcano plots (Figure S15B–E, Supporting Information). The number of DEGs between APAP model and control mice was 6495, while the DEGs in NAC, DNase I, and combination therapy compared to APAP model were 1285, 821, and 1436, respectively. In Gene Ontology (GO) enrichment analysis, the comparison between APAP model and control mice confirmed that the activation of immune response and the impairment of oxidative balance together played important roles in AILI (Figure S15F, Supporting Information). Further analysis indicated that the MAPK, NF‐*κ*B, TNF, and TGF signaling pathways were highly associated with the therapeutic mechanisms of DNase I (Figure S15H, Supporting Information). In addition to those pathways, oxidation‐production process, cellular response to hydrogen peroxide, and glutamine metabolism process showed enrichment during combined therapy (**Figure** **6**A). Pathway analysis comparing the combination therapy with APAP model confirmed that the reduction of inflammation and oxidative stress was involved in the combined treatment of AILI.

Accordingly, RNA sequencing and qPCR confirmed the change on the expression of genes related to oxidative stress and inflammation. Several inflammation and oxidative stress related genes, including *Tnf*, *Il1β*, *Mmp3*, *Sphk1*, *Alox12*, and *Nqo1*, were significantly downregulated after the combination therapy (Figure 6B–D). As determined by liver IHC, combined treatment consistently decreased the expression of classic inflammation markers TNF‐*α* and TGF‐*β*, and oxidative stress marker 3‐nitrotyrosine (3‐NT) (Figure 6E and Figure S16A–D, Supporting Information). Likewise, single therapy alone showed inferior results in inflammation and oxidative stress when compared with combination therapy.

Given cfDNA was able to be specifically sensed by TLR9 to trigger immune responses, we determined the expression of TLR9 in liver by IHC. The expression of TLR9 was upregulated in the liver during AILI, while DNase I treatment resulted in a significant TLR9 decrease by cfDNA degradation (Figure 6E,F and Figure S16E, Supporting Information). Consistently, both DNase I and combination therapy reduced the TLR9 expression in hepatic neutrophils (Figure 6G and Figure S16F, Supporting Information). We further confirmed that DNase I blocked CpG ODN 2006‐triggered TLR9 activation in HEK‐Blue TLR9 reporter cells, while the combination of DNase I and NAC was more effective in the blockage (Figure 6H). Together, the combination of DNase I and NAC reduced the expression and activation of TLR9 to ameliorate excessive liver inflammation, further indicating that neutrophils may serve as one of crucial sensors and effectors of cfDNA during AILI. In addition, the combination therapy reduced inflammation and oxidative stress in AILI to rescue liver damage.

## 3 Discussion

In the clinic, the circulating paracetamol concentration is a pivotal parameter for the diagnosis and prognosis of AILI, but the definite time of APAP ingestion must be known.^[^ ^12^ ^]^ Although ALT and AST provide crucial information for liver damage, they have limited sensitivity and may be unreliable because their increase may be delayed.^[^ ^3^, ^13^ ^]^ Therefore, a more accurate biomarker for prognosis of AILI is urgently needed. It is well known that mitochondrial and chromatin damage is involved in AILI, and a large amount of DAMPs is released from damaged liver cells.^[^ ^10^, ^14^ ^]^ Such circulating danger molecules including HMGB1, keratin 18, and mtDNA have been demonstrated to correlate with later injury or death in AILI.^[^ ^10^, ^13^ ^]^ In particular, higher circulating cfDNA levels have been reported in patients with AILI,^[^ ^10^ ^]^ but the mechanisms behind this and its potential as mechanistic biomarker or as therapeutic target have not been elucidated so far. Deep understanding of the process of APAP hepatotoxicity from damage to inflammation will help us explore new biomarkers and targets for precision‐guided AILI management.

The objective of the current study was to gain further insight into the potential role of circulating cfDNA in liver damage and inflammation in a temporal manner and provide evidence for the substantial value of cfDNA as a promising mechanistic biomarker and target. First, we detected significantly higher circulating cfDNA in DILI and ALD patients. Using the multi‐timepoint analysis on APAP‐challenged mice, we provided evidence for the crucial role of circulating cfDNA in the development and progression of AILI, which correlated with hepatic damage and inflammation. In previous studies, circulating HMGB1 exhibited strong relation to ALT.^[^ ^13^ ^]^ Compared with HMGB1, our current findings demonstrated that circulating cfDNA exhibited stronger correlation with damage markers ALT, AST, and necrosis area, along with inflammation marker TNF‐*α* at each timepoint. We found the first peak of circulating cfDNA at 18 h after APAP challenge, which might be attributed to liver and local immune cells under oxidative stress. The decreased cfDNA level at 24 h post APAP overdose might be explained by the degradation of cfDNA by DNase or metabolic clearance by phagocytes. Although the cfDNA level decreased at 24 h, it was still much higher than the normal group. Interestingly, the second peak of circulating cfDNA was observed at 36 h, which might be mainly derived from leukocytes and intestinal microflora. A previous study revealed that contributors of circulating cfDNA in healthy volunteers mainly came from granulocytes, monocytes, lymphocytes, and erythrocyte progenitors. In the case of septic patients with liver damage, the main contributors of circulating cfDNA were hepatocytes and leukocytes,^[^ ^15^ ^]^ which could provide further insights. Additional investigation of the time‐related cfDNA source will be needed to identify its cell, tissue, and organ origin by genome‐wide methylation profiling.

In AILI model with different grades, we found that cfDNA provided a better picture on the severity of liver damage. Recent research showed that the severity of COVID‐19 infection and non‐alcoholic fatty liver disease (NAFLD) severity was associated with higher circulating cfDNA levels.^[^ ^16^ ^]^ NETs, one form of cfDNA, were also elevated in patients with non‐alcoholic steatohepatitis.^[^ ^17^ ^]^ Similarly, higher cfDNA in patients with ALF is associated with poor outcome.^[^ ^3b^ ^]^ Consistent with this, elevated cfDNA level in our lethal AILI model was also strongly associated with mortality. Given the diverse origin of cfDNA, the specificity may be somewhat unsatisfactory while the sensitivity is comparable to ALT during the prediction. Such a high sensitivity of cfDNA may be attributed to its short half‐life (estimated 15–60 min)^[^ ^18^ ^]^ compared with aminotransferase. Nevertheless, we found the combination of cfDNA and ALT could predict animal death better than ALT alone in AILI, indicating its potential in the clinic. Although our study provided significant support for the utility of cfDNA profiling as viable biomarker to aid the detection and prognosis prediction of AILI, it is difficult to predict which patients will have high risk of mortality in the clinic, so more patients are required to validate our findings and confirm the best timing and threshold of circulating cfDNA to predict the outcome of AILI.

We also found the causative role of cfDNA by its scavenge or addition in AILI, but how cfDNA elicited liver damage is worth pondering. Neutrophils showed a rapid amplification in both peripheral and liver immune cells during AILI, while its depletion led to reduction of circulating cfDNA levels and liver damage. Correspondingly, the adoptive transfer of neutrophils resulted in adverse consequences. In addition, DNase I treatment not only decreased the population of hepatic neutrophils, but also reversed the neutrophil‐exacerbated liver injury. These findings collectively indicated a positive feedback loop between cfDNA and neutrophils during AILI. Increasing evidence demonstrated that excessive activation and recruitment of neutrophils could cause additional liver injury by producing abundant pro‐inflammatory cytokines and DAMPs, such as NETs, which contributes to the elevation of circulating cfDNA. Since cfDNA is sensed by neutrophils and serves as bridge to concatenate damage and inflammation,^[^ ^11^ ^]^ the cfDNA‐exacerbated neutrophils activation boosts inflammatory cascades for further liver damage. Thus, we speculated that neutrophils were not only one of the key target cells of cfDNA, but also became a significant origin of circulating cfDNA in AILI. Some research also indicated the complex function of neutrophils in different stages of the AILI, which might promote the repair and regeneration in late stage.^[^ ^19^ ^]^ This should be elucidated in further studies. The heterogeneity of macrophages decided that they performed in a sophisticated manner during AILI.^[^ ^20^ ^]^ The depletion of KCs impaired hepatic innate immunity. Thereafter, large numbers of monocytes rapidly infiltrated into liver and differentiated into Ly6C^hi^ and Ly6C^lo^ macrophages.^[^ ^20^, ^21^ ^]^ Therefore, when macrophages were depleted indiscriminately in AILI mice, liver damage was aggravated.

The abovementioned findings suggested the mechanistic role for circulating cfDNA in the prognosis of AILI, simultaneously, constituted a strong rationale to test its therapeutic potential. DNase I has been widely proved to hold therapeutic value in sepsis, ischemia reperfusion injury, and NAFLD by direct degradation of cfDNA.^[^ ^8^, ^11^, ^17^ ^]^ Consistently, we demonstrated the protective effect of DNase I in AILI model with different degrees. Scavenging cfDNA in AILI suppressed the recruitment and activation of neutrophils, alleviating hepatic injury mainly through reduction of inflammation. Since NAC could reduce liver injury of APAP overdose in clinic by alleviating oxidative stress, it inspired us to integrate NAC with DNase I as a combination strategy of antioxidant and anti‐inflammation. We demonstrated the advantages of combined therapy compared with monotherapies. By systematically studying the mechanisms of combination therapy, we revealed the mitigation of inflammation and oxidative damage after treatment. In particular, treatment containing DNase I lowered TLR9 expression in liver and hepatic neutrophils and inhibited the activation of TLR9, which could be responsible for the suppression of inflammation. Since TLR9 was extensively expressed on hepatic stellate cells, macrophages, and sinusoidal endothelial cells,^[^ ^22^ ^]^ deciphering key cfDNA sensing cells might provide new targets for AILI management. Besides TLR9, other intracellular cfDNA sensors including inflammasomes or cGAS,^[^ ^23^ ^]^ may also be involved in inflammation during AILI. cfDNA is a pro‐inflammatory TLR9 agonist and activates MAPKs and NF‐*κ*B to trigger inflammatory responses.^[^ ^24^ ^]^ Therefore, further investigation of the mechanisms of cfDNA in the inflammatory response is highly needed.

In summary, the current study revealed higher circulating cfDNA levels in DILI and ALD patients and systematically illustrated that circulating cfDNA displayed a time‐dependent pattern in the interaction between liver damage and inflammation during AILI. The potential of circulating cfDNA as a biomarker and the mechanistic principles were extensively validated by parallel studies in mice. cfDNA could be considered as a mechanistic indicator and used alongside established ALT to predict the risk of mortality during APAP overdose. Neutrophils were important sensors and effector cells of cfDNA in AILI. cfDNA could amplify inflammation and damage at least partly through neutrophils. Deciphering the crucial role of circulating cfDNA on bridging liver damage and inflammation defines its merits as a therapeutic target in AILI. After demonstrating that cfDNA could be an attractive target to promote the resolution of inflammation, we developed a new combination strategy of DNase I and NAC by targeting both inflammatory response and oxidative damage during AILI. Our findings confirmed the potential of circulating cfDNA as a new predictive and therapeutic target for APAP hepatotoxicity. As a result, sophisticated combination therapy targeting both inflammation and oxidative damage may be a fruitful direction of AILI, along with other liver injuries and diseases.

## 4 Experimental Section

### Patients

A prospective study was conducted and 40 patients with DILI, 40 patients with ALD, and 40 HV were recruited from August 2021 until January 2022 from the Third Affiliated Hospital of Sun Yat‐sen University ([2022]02‐197‐01). The inclusion and exclusion criteria were defined according to the Guidelines for the diagnosis and management of DILI and Guidelines of prevention and treatment for ALD: a 2018 update published by the Chinese Society of Hepatology. The collection of samples was performed under the approval of the Ethics Committee at the Third Affiliated Hospital of Sun Yat‐sen University and all subjects provided written informed consent. Following isolation of plasma, samples were frozen at −80 °C until analysis.

### Acetaminophen‐Induced Liver Injury Model

Male C57BL/6J wild‐type mice (5–7 weeks) were purchased from Hunan SJA Laboratory Animal Limited Company and acclimatized for 1 week before experiments. All animal procedures were approved by the Institutional Animal Care and Use Committee of South China University of Technology (Guangzhou, China). After fasting for 16 h, APAP (Sigma‐Aldrich) was given at dose of 250 mg kg^−1^ (mild), 450 mg kg^−1^ (moderate), and 550 mg kg^−1^ (severe) by a single i.p. to induce AILI.

### The Temporal Pattern of Liver Injury

In the moderate AILI model, blood and liver samples were collected at 0, 2, 6, 9, 12, 18, 24, 36, and 48 h after APAP administration. Then the levels of circulating ALT, AST, cfDNA, HMGB1, and TNF‐*α* were detected. The necrosis area in the hematoxylin and eosin staining of livers was calculated.

### Purification and Quantification of cfDNA

QIAamp DNA Blood Mini Kit (Qiagen) was utilized for purification of cfDNA from serum or plasma according to the manufacturer's instructions. The levels of cfDNA were measured using Quant‐iT PicoGreen dsDNA Assay Kit (Thermo Fisher) by detecting the fluorescence (Ex: 480 nm, Em: 520 nm).^[^ ^25^ ^]^

### DNase I, CpG ODN 1826, and NAC Treatment

NAC (150 mg kg^−1^, i.p., Sigma‐Aldrich) was administered after 3 h (or 10 h) and DNase I (80 U/dose, Sigma‐Aldrich) was administered by i.v. at 3 and 10 h (or 10 h) after APAP challenge. CpG ODN 1826 (0.5 mg kg^−1^, i.v., Sangon Biotech) was given at 3 h after APAP. Animals were sacrificed at 12 or 24 h to detect related parameters. In the severe model, the survival rates were monitored for 96 h.

### Flow Cytometry

After incubating with FcBlock (TruStain FcX anti‐mouse CD16/32, Biolegend) to block non‐specific binding, the isolated non‐parenchymal liver cells and peripheral blood leukocytes were stained with fluorochrome‐conjugated monoclonal antibodies at 4 °C for 30 min in the dark and then washed with PBS. Cells were fixed and permeabilized before the staining of TLR9. Monoclonal antibodies included anti‐CD45.2 APC/Cy7 (Biolegend), anti‐CD11b BV510 (BD), anti‐F4/80 PE/Cy7 (Biolegend), anti‐F4/80 BV785 (Biolegend), anti‐Gr‐1 PE‐CF594 (BD), anti‐NK1.1 FITC (Biolegend), anti‐CD3 BV421 (Biolegend), anti‐CD4 BUV563 (BD), anti‐CD8a BV711 (Biolegend), anti‐CD19 PE/Cy5 (Biolegend), anti‐I‐A/I‐E Alexa Fluor 700 (Biolegend), and anti‐CD289 (TLR9) FITC (eBioscience). Data were collected using a flow cytometer (LSRFortessa or FACS Aria, BD) and analyzed using FlowJo V10 software.

### The Depletion of Neutrophils and Macrophages

For the depletion of neutrophils, anti‐mouse Ly6G/Ly6C antibody (Bioxcell) at 100 µg/dose (i.p.) was administrated at 24 and 4 h before the APAP challenge. Rat IgG2b (Bioxcell) was used as isotype control. For macrophages depletion, 250 µL Clodronate liposomes (Liposoma) or control liposomes (Liposoma) (i.p.) was given at 48 h before APAP challenge. The depletion was verified by flow cytometry (FACS Aria, BD).

### Adoptive Transfer of Neutrophils

Mouse neutrophils were isolated and purified from bone marrow of tibias and femurs as described.^[^ ^26^ ^]^ Before APAP challenge, 6 × 10^6^ neutrophils were adoptively transferred into mice by i.v. Animals were sacrificed at 24 h to detect related parameters.

### TLR Activation Assays In Vitro

To determine the inhibition of TLR9 activation by DNase I and NAC, HEK‐Blue TLR9 reporter cells at a density of 8 × 10^4^ per well in a 96‐well plate were incubated with CpG ODN 2006 (1.8 µg mL^−1^), DNase I (5 U mL^−1^), and NAC (10 mm). After 24 h, 20 µL supernatants were collected and incubated with 180 µL QUANTI‐Blue (Invivogen) for 2 h. The embryonic alkaline phosphatase (SEAP) activity was determined by measuring the optical density at 620 nm (OD_620_) to quantify the activation of TLR9.

### Transcriptome Sequencing Analysis

Total RNA was extracted from mouse liver samples with TRIzol reagent (Invitrogen) according to the manufacturer's instructions. Then total RNA was qualified and quantified using a Nano Drop (2000, Thermo Fisher) and Agilent bioanalyzer (2100, Thermo Fisher). Subsequently, the RNA purification, reverse transcription, end repair, library construction, and sequencing were performed.

For bioinformatics analysis, the expression level of each transcript was calculated according to the fragments per kilobase of exon per million mapped reads (FPKM) method. Differential expression analysis was performed using the DEGseq with |log2 FC| ≥ 0.5 and *Q* value ≤ 0.001 and GO enrichment analysis of annotated DEGs was performed by Phyper based on hypergeometric test. The significant levels of terms and pathways were corrected by *Q* value with a rigorous threshold by Bonferroni.

### Statistical Analysis

All data were presented as mean ± SD. The Shapiro–Wilk test was used to detect the normal distribution of data. Unpaired *t* test, ordinary one‐way ANOVA with Tukey's Multiple Comparison test, and Kruskal–Wallis's test were used to analyze the differences between groups. Detailed sample size was shown in the corresponding figure legends. Differences were considered significant when *p* < 0.05. Associations among variables were determined using Spearman correlation analysis. Statistical analysis was performed in GraphPad Prism 8.3.

The ROC curve analysis was performed using Statistic Package for Social Science 23 (SPSS 23). The area under curve was determined and compared by DeLong's test. Differences were considered significant when *p* < 0.05.

The IDI, risk assessment plots, and NRI were utilized to determine the added value of cfDNA on basal ALT in predicting outcome.^[^ ^3a^ ^]^ All analyses were performed using R 4.1.0 (The R Foundation for Statistical Computing).

For further details regarding the materials used, please refer to the Supporting Information and CTAT table.

## Conflict of Interest

The authors declare no conflict of interest.

## Author Contributions

M.S. and P.C. contributed equally to this work. M.S., P.C., C.G., X.H., and T.S. performed the experiments. K.X., Z.Z., and Y.J. performed the data analysis. X.Z., Q.Z., and Y.T. were responsible for samples collection. M.S. and P.C. performed data interpretation and drafted manuscript. M.L., K.W.L., and D.S. conceived and supervised the project, and revised the manuscript.

## Supporting information

Supporting Information

## Data Availability

The data that support the findings of this study are available from the corresponding author upon reasonable request.
