# Supplementary material for: Circulating Cell‐Free DNAs as a Biomarker and Therapeutic Target for Acetaminophen‐Induced Liver Injury
Source: Adv Sci (Weinh). 2023 Apr 10;10(16):2206789. doi: 10.1002/advs.202206789 (PMC10238175; doi:10.1002/advs.202206789)
Supplement: Supplementary file 1 — Supporting Information [file ADVS-10-2206789-s001.pdf]

Supporting Information

Circulating Cell-free DNA as a Biomarker and Therapeutic Target for Acetaminophen-Induced Liver Injury

Madi Sun, Peiyu Chen, Kai Xiao, Xiang Zhu, Zhibin Zhao, Chenyang Guo, Xuan He, Tongfei Shi, Qingguo Zhong, Yong Jia, Yu Tao, Mingqiang Li, Kam W. Leong*, and Dan Shao**

M. Sun, P. Chen, C. Guo, X. He, T. Shi, Prof. D. Shao
School of Biomedical Sciences and Engineering, South China University of Technology, Guangzhou International Campus, Guangzhou, Guangdong 510630, China
E-mail: stanauagate@outlook.com; shaodan@scut.edu.

M. Sun, P. Chen, K. Xiao, C. Guo, X. He, T. Shi, Prof. D. Shao
National Engineering Research Center for Tissue Restoration and Reconstruction, South China University of Technology, Guangzhou International Campus, Guangzhou, Guangdong 510630, China

K. Xiao, Dr. Z. Zhao
School of Medicine, South China University of Technology, Guangzhou International Campus, Guangzhou, Guangdong 510006, China

Dr. Y. Jia
School of Nursing, Jilin University, Changchun, Jilin 130021, China

X. Zhu, Q. Zhong, Y. Tao, Prof. M. Li
Laboratory of Biomaterials and Translational Medicine, The Third Affiliated Hospital Sun Yat-sen University, Guangzhou, Guangdong 510006, China
E-mail: limq567@mail.sysu.edu.cn

Prof. K. W. Leong
Department of Systems Biology, Columbia University, New York, NY 10032, USA
E-mail: kam.leong@columbia.edu

Prof. D. Shao
Guangdong Provincial Key Laboratory of Biomedical Engineering, Key Laboratory of Biomedical Materials and Engineering of the Ministry of Education, South China University of Technology
Guangzhou, Guangdong 510006, China

Experimental Section

1. Serum analyses

The mice were sacrificed and serum was collected. Alanine aminotransferase (ALT), aspartate aminotransferase (AST), urea (UREA), creatinine (CRE) and creatine kinase (CK) were evaluated using automatic biochemical analyzer (3100, Hitachi). High mobility group box-1 protein (HMGB1), tumor necrosis factor-α (TNF-α), interleukin-6 (IL-6) and macrophage chemoattractant protein-1 (MCP-1) in serum were quantified by ELISA (Elabscience).

2. Isolation of peripheral blood leukocytes

For the isolation of peripheral blood leukocytes, mice were anesthetized to obtain the whole blood and the erythrocytes were lysed to isolate leukocytes.

3. Isolation of non-parenchymal liver cells

For non-parenchymal liver cells (NPC) isolation, the portal vein was cannulated and the liver was perfused with 0.5 mM EGTA (5 mL/min, 37 °C preheating) to flush the blood. Then, the liver was perfused with 0.075 % collagenase type IV (Perfusate, 3 mL/min, 37 °C preheating, Sigma-Aldrich) for 4 min, after which it was excised, placed in 87.5 % DMEM (Gibco) + 12.5 % Perfusate and brushed into homogenates. The homogenates were digested for 20 min in a shaking incubator (THZ-100, Bluepard) at 37 °C at 120 rpm and filtered through 100-mesh nylon screen to obtain single-cell suspensions. After centrifugation (50 g, 5 min) twice, the supernatant was

centrifuged for 10 min at 500 g, and then the precipitate was resuspended in 25 % isotonic Percoll and layered over 70% isotonic Percoll (Biosharp). After which the NPCs were separated from the hepatocytes by differential centrifugation (800 g for 15 min) at room temperature.

4. CpG-induced liver injury model and treatment

After 1 h of sensitization with D-GalN (800 mg/kg, i.p., Adamas), mice were administered with CpG ODN 1826 (0.75 mg/kg, i.p.) to induce acute liver injury. The depletion of neutrophils or macrophages were achieved by anti-mouse Ly6G/Ly6C antibody or Clodronate liposomes as previously described. DNase I was administered (80 U/dose, i.v.) at 3 and 10 h after CpG-challenge. After 24 h the mice were sacrificed to detect the levels of cfDNA and ALT.

5. The isolation and purification of mouse bone marrow-derived neutrophils

Mice were euthanized then doused in 75% ethanol. The tibias and femurs were removed from both legs entirely and bone marrow was flushed from the bones. Then the bone marrow fluid was filtered through 100-mesh nylon screen to obtain single-cell suspensions. After centrifugation (230 g, 6 min), the precipitate was resuspended in DMEM. 2.5 mL 72% Percoll, 2 mL 62% Percoll and 2 mL 52% Percoll were layered in a 15-ml conical tube. Overlay the bone marrow cell suspension on the top and the neutrophils were separated by differential centrifugation (1545 g for 30 min) at room temperature. Count the neutrophils and determine their purity by flow cytometry.

6. The optimization of drug administration in vivo

In mild APAP model, NAC (150 mg/kg, i.p., Sigma-Aldrich) was administered at 1/12 h, 3/12 h, 6/12 h or 3 h after APAP challenge to determine the administration time. Different doses of DNase I (24, 40, 56, 80, 160 and 480 U/dose, Sigma-Aldrich) was administered by intravenous injection (i.v.) at 6 and 12 h after APAP challenge. For combination therapy of NAC and DNase I, NAC (150 mg/kg, i.p.) was administered at 3 h and different doses of DNase I (40, 80, 120 and 160 U/dose) was administered at 3 h and 10 h after APAP challenge. After 24 h, the mice were sacrificed and serum was collected to detect the levels of ALT and AST.

7. Liver homogenate and redox factors

Liver tissue was homogenized in normal saline to prepare 10% liver homogenate. The supernatant was collected after centrifugation (4 ℃, 3000 rpm, 10 min). Total protein was measured by BCA protein assay kit (Thermo Fisher). The liver homogenate was diluted to different concentrations for following detection. The activity GSH-Px, and the levels of MDA were determined with assay kits, respectively (Nanjing Jiancheng).

8. Histopathological assessment and immunohistochemistry

Pathological sections of liver were stained with hematoxylin and eosin (H&E). The necrosis area was measured using Image J and presented in the ratio of necrotic

area to total area.

For immunohistochemistry, after deparaffinized, rehydrated and heat-induced epitope retrieval, the slices were incubated in 3% H_2O_2, and then in 3% serum to block endogenous peroxidase activity and nonspecific binding. Then the primary antibodies against TLR9 (ab37154, 1:200; Abcam), Ly6G (ab238132, 1:200; Abcam), TNF-α (ab9739, 1:200; Abcam), 3-NT (ab61392, 1:100; Abcam) and TGF-β (ab215715, 1:200; Abcam) were incubated with slices at 4 °C overnight. The slices were further incubated with HRP-labelled secondary antibody (anti-mouse or anti-rabbit) and diaminobenzidine (DAB) detection solution. Then, the slices were counterstained with hematoxylin, dehydrated and mounted. Evaluation of IHC was performed by the H-score system. The staining intensity was defined as: 0, no staining; 1+, weak; 2+, moderate and 3+, strong staining and the positively stained area was estimated as percent (0 %–100 %). H-score was generated by multiplying the percentage of positive area and staining intensity score (H-score = 3 × the strong staining percentage + 2 × the moderate staining percentage + 1 × the weak staining percentage + 0 × no staining percentage).

TdT-mediated dUTP Nick-End Labeling (TUNEL) staining was performed using One Step TUNEL Apoptosis Assay Kit (Beyotime) according to the manufacturer's instructions. The nucleus is labeled with DAPI. TUNEL staining was quantified by the percentage of positive cells.

9. In vivo biosafety profile

Mice were administrated with NAC and DNase I as mentioned previously and the body weight was recorded once a week. After 28 days, the serum was collected to perform analyses. The heart, liver, spleen, lung and kidney were collected for histopathological examination.

10. Quantitative real-time PCR analysis

Total RNA was isolated from mouse liver samples with TRIzol reagent (Invitrogen) and reverse transcription of 1 μg RNA was performed using the PrimeScript™ RT reagent Kit with gDNA Eraser (Takara) following the manufacturer's protocol. The resulting cDNAs were amplified by PCR with TB Green® Premix Ex Taq™ II Kit (Takara) in a real-time PCR system (LightCycler® 96 instrument, Roche). The amplified transcripts relative to the GAPDH endogenous control were quantified using the comparative $2^{-\Delta\Delta Ct}$ method.

The following primer sequences synthesized by Sangon Biotech were used:

**Gene**	**Forward primer (5'- to -3')**	**Reverse primer (5'- to -3')**
Nqo1	TTC TGT GGC TTC CAG GTC TT	TCC AGA CGT TTC TTC CAT CC
Sphk1	GGC TCT GCA GCT CTT CCA GAG	CTC CTC TGC ACA CAC CAG CTC
Alox12	GAT CAC TGA AGT GGG GCT GT	CAC ACA TGG TGA GGA AAT GG
Tnf	CAC GTC GTA GCA AAC CAC C	TGA GAT CCA TGC CGT TGG C
Il1b	AGA GCC CAT CCT CTG TGA CTC A	TGC TTG GGA TCC ACA CTC TCC A
Mmp3	GTC CCT CTA TGG AAC TCC CAC	AGT CCT GAG AGA TTT GCG CC
GAPDH	TGT GTC CGT CGT GGA TCT GA	CCT GCT TCA CCA CCT TCT TGA T

11. CTAT table

**Reagent**	**Supplier**	**Cat no.**
Acetaminophen	Sigma-Aldrich	A7085
N-Acetyl-L-cysteine	Sigma-Aldrich	A7250
Deoxyribonuclease I	Sigma-Aldrich	DN25
Collagenase Type IV	Sigma-Aldrich	C5138
Clodronate liposomes	Liposoma	P67E1221
Control liposomes	Liposoma	P69E1221
Percoll	biosharp	BS909
D-Chondrosamine Hydrochloride	Adamas-beta	01035162
Red Blood Cell Lysis Buffer	Beyotime	C3702
Dulbecco's Modified Eagle Medium	Gibco	A4192101
TRIzol™ LS Reagent	Invitrogen	10296010
QIAamp DNA Blood Mini Kit	Qiagen	51106
QuantiT™ PicoGreen™ dsDNA Assay Kit	Thermo Fisher	P11496
Mouse HMGB-1 (High Mobility Group Pritein B1) ELISA Kit	Elabscience	E-EL-M0676c
Mouse TNF-α (Tumor Necrosis	Elabscience	E-EL-M0049c

Factor Alpha) ELISA Kit		
Mouse IL-6 (Interleukin 6) ELISA Kit	Elabscience	E-EL-M0676c
Mouse MCP-1 (Moncyte Chemotactic Protein 1) ELISA Kit	Elabscience	E-EL-M3001
Glutathione Peroxidase (GSH-Px) assay kit (Colorimetric method)	Nanjing Jiancheng	A005-1
Malondialdehyde (MDA) assay kit (TBA method)	Nanjing Jiancheng	A003-1
BCA Protein Assay Kit	Thermo Fisher	23225
Fixation/Permeabilization Kit	BD	554714
PrimeScript™ RT reagent Kit with gDNA Eraser (Perfect Real Time)	Takara	RR047A
TB Green® Premix Ex Taq™ II (Tli RNaseH Plus)	Takara	RR820B
Hematoxylin and Eosin Staining Kit	Beyotime	C0105S
One Step TUNEL Apoptosis Assay Kit	Beyotime	C1086
UltraSensitive™ SP（mouse/rabbit）IHC Kit	Fuzhou Maixin	KIT-9710

DAB Plus Kit	Fuzhou Maixin	DAB-2031
**Antibody**	**Supplier**	**Cat no.**
Anti-mouse Ly6C/Ly6G	Bioxcell	BE0320
Rat IgG2b isotype control	Bioxcell	BE0090
TruStain FcX™ (anti-mouse CD16/32) Antibody	Biolegend	101320
FITC anti-mouse NK 1.1 Antibody	Biolegend	108706
PE/Cyanine7 anti-mouse F4/80 Antibody	Biolegend	123114
PE/Cyanine5 anti-mouse CD19 Antibody	Biolegend	115510
APC/Cyanine7 anti-mouse CD45.2 Antibody	Biolegend	109824
Alexa Fluor® 700 anti-mouse I-A/I-E Antibody	Biolegend	107622
Brilliant Violet 421™ anti-mouse CD3 Antibody	Biolegend	100228
Brilliant Violet 711™ anti-mouse CD8a Antibody	Biolegend	100748
Brilliant Violet 785™ anti-mouse F4/80 Antibody	Biolegend	123141
PE-CF594 Rat Anti-Mouse Ly-	BD	562710

6G and Ly-6C		
V500 Rat anti-CD11b	BD	562127
BUV563 Rat Anti-Mouse CD4	BD	565709
FITC CD289 (TLR9) Monoclonal Antibody	Invivogen	11-9093-82
Anti-TLR9 antibody	Abcam	ab37154
Anti-Ly6g antibody	Abcam	ab238132
Anti-TNF alpha antibody	Abcam	ab9739
Anti-3-Nitrotyrosine antibody	Abcam	ab61392
Anti-TGF beta 1 antibody	Abcam	ab215715
**Cell line**	**Supplier**	**Cat no.**
HEK-Blue™ TLR9 reporter cells	Invivogen	hkb-htlr9
**Name**	**Supplier**	**Sequence (5' to 3')**
CpG ODN 1826	Sangon Biotech	TCC CTG ACG TTC CTG ACG TT
CpG ODN 2006	Sangon Biotech	TCG TCG TTT TGT CGT TTT GTC GTT

Results

Table S1.

Demographic data of patients and healthy volunteer

	All patients and volunteers (n = 120)	Healthy volunteer (n = 40)	Liver injury patients (n = 80)	
			Drug-induced liver injury patients (n = 40)	Alcoholic liver disease patients (n = 40)
Females (%)	44 (36.67%)	21 (52.50%)	22 (55.00%)	1 (2.50%)
Age (years) AVE(Range)	43 (18-84)	31 (18-53)	54 (18-84)	44 (24-65)
ALT (U/L) AVE(Range)	65.93 (3-550)	19.43 (6-50)	138.75 (13-550)	39.60 (3-297)
cfDNA (ng/mL) AVE(Range)	154.02 (4.14-1075.21)	61.07 (4.14-218.67)	208.10 (25.36-629.62)	192.89 (22.46-1075.21)

Table S2.

Comparison of the ability of ALT and cfDNA (12 h after APAP) to predict death (n=20)

	AUROC (95% CI)	Best cut-off	Sensitivity (95% CI)	Specificity (95% CI)	Likelihood ratio
ALT (n = 20)	0.8958 (0.7581-1.000) (P=0.0034)	> 5340 U/L	75.00 % (46.77-91.11 %)	100.0% (67.56-100.0 %)	-
cfDNA (n = 20)	0.9479 (0.8527-1.000) (P=0.0009)	> 17.66μg /mL	91.67 % (64.61-99.57 %)	87.50% (52.91-99.36 %)	7.333
ALT + cfDNA (n = 20)	0.9583 (0.8786-1.000) (P=0.0007)	-	91.67 % (64.61-99.57 %)	87.50% (52.91-99.36 %)	7.333

AUROC, the area under the receiver-operating characteristic curve.

Table S3.

IDI and NRI of cfDNA at presentation for the prediction of death

Baseline Model	Additional variable	Event (death)	IDI (95%CI)	P-value of IDI	NRI (continuous) (95% CI)	P-value of NRI
ALT (n = 20)	cfDNA (n = 20)	12	0.2158 (0.0426-0.3889)	0.01461	1.0833 (0.38-1.7866)	0.00254
ALT (n = 40)	cfDNA (n = 40)	18	0.1928 (0.0601-0.3255)	0.00441	0.8586 (0.3047-1.4125)	0.00238

IDI, integrated discriminatory improvement; NRI, net reclassification improvement.

Table S4.

Comparison of the ability of ALT and cfDNA (12 h after APAP) to predict death (n=40)

	**AUROC (95% CI)**	**Best cut-off**	**Sensitivity (95% CI)**	**Specificity (95% CI)**	**Likelihood ratio**
ALT (n = 40)	0.8712 (0.7499-0.9925) (P < 0.0001)	> 5340 U/L	77.78 % (54.79-91.00 %)	95.45 % (78.20-99.77 %)	17.11
cfDNA (n = 40)	0.9394 (0.8700-1.000) (P < 0.0001)	> 19.15μg/mL	94.44 % (74.24-99.72 %)	81.82% (61.48-92.69 %)	5.194
ALT + cfDNA (n = 40)	0.9394 (0.8710-1.000) (P < 0.0001)	-	94.44 % (74.24-99.72 %)	81.82% (61.48-92.69 %)	5.194

AUROC, the area under the receiver-operating characteristic curve.

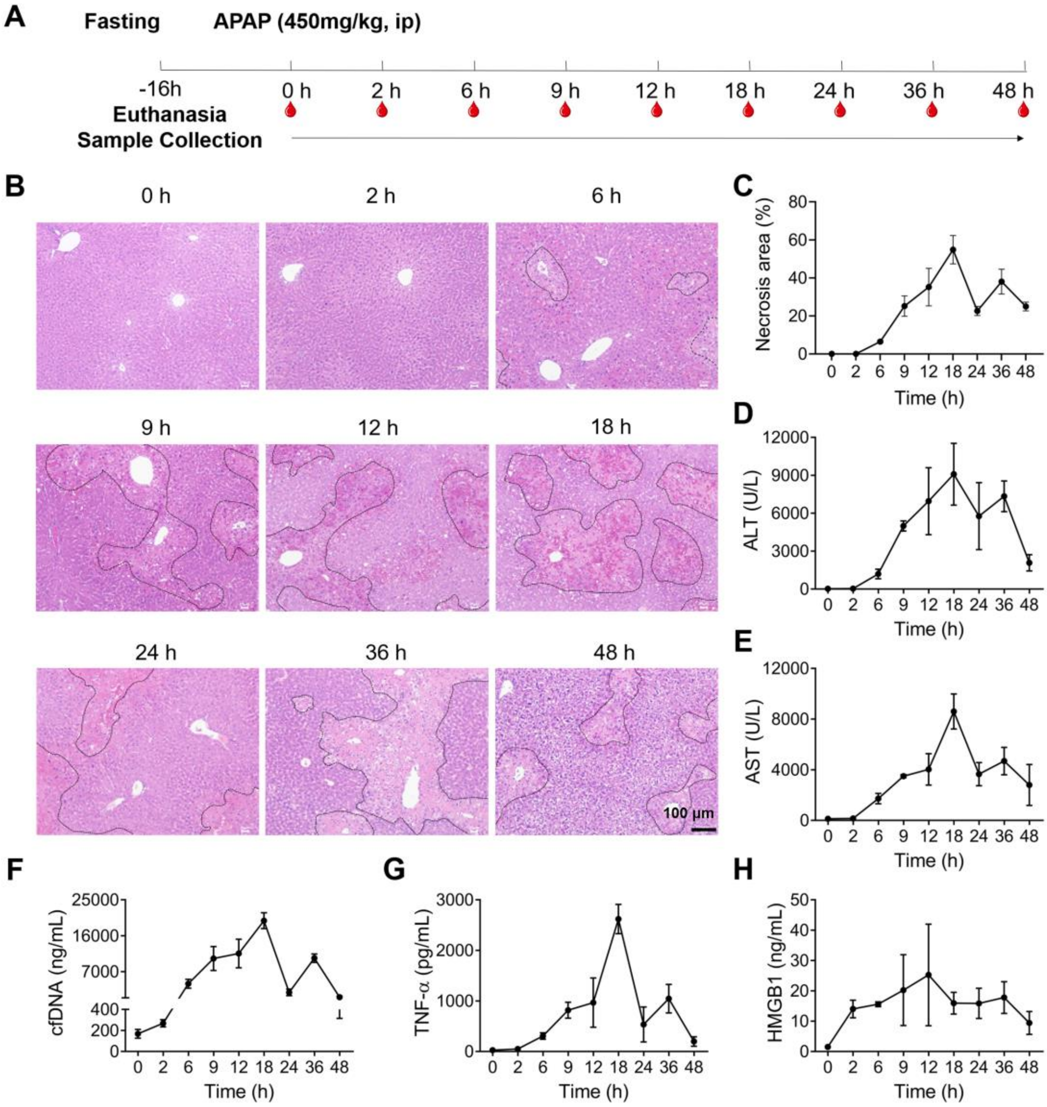

Figure S1. The temporal pattern of parameters related to liver damage and inflammation in AILI (450 mg/kg). (A) Scheme of the experimental design. (B) Representative H&E staining of liver at different time points. Original magnification, ×100; Scale bar, 100 μm. (C-H) The levels of liver necrosis area, circulating ALT, AST, cfDNA, TNF-α and HMGB1 were measured at different time points after APAP challenge (n = 3-5 for each time point). Data are expressed as means ± SD.

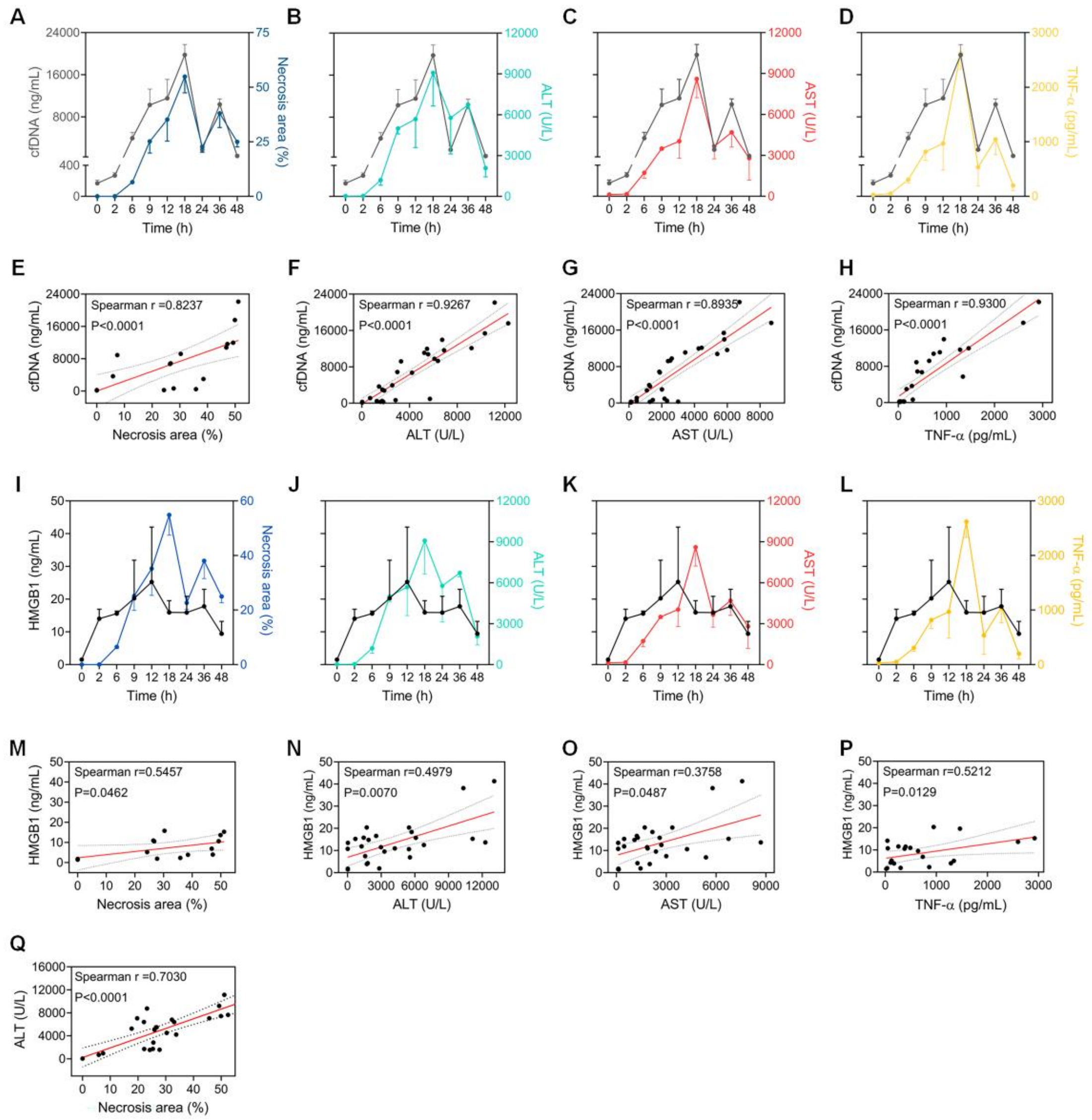

Figure S2. Circulating cfDNA exhibits stronger correlation with liver damage and inflammation factors compared with HMGB1 in AILI (450 mg/kg). (A-D) The data fitting between the levels of circulating cfDNA with liver necrosis area, circulating ALT, AST and TNF-α (n = 3-5 for each time point). Data are expressed as means ± SD. (E-H) Correlation between cfDNA level and liver necrosis area (n = 18), circulating ALT (n = 33), AST (n = 33) and TNF-α (n = 25). (I-L) The data fitting between the levels of circulating HMGB1 with liver necrosis area, circulating ALT, AST and TNF-α. Data are expressed as means ± SD (n = 3-5 for each time point). (M-P) Correlation between HMGB1 with liver necrosis area (n = 14), circulating ALT (n = 28), AST (n = 28) and TNF-α (n = 22). (Q) Correlation between ALT with liver necrosis area (n = 26).

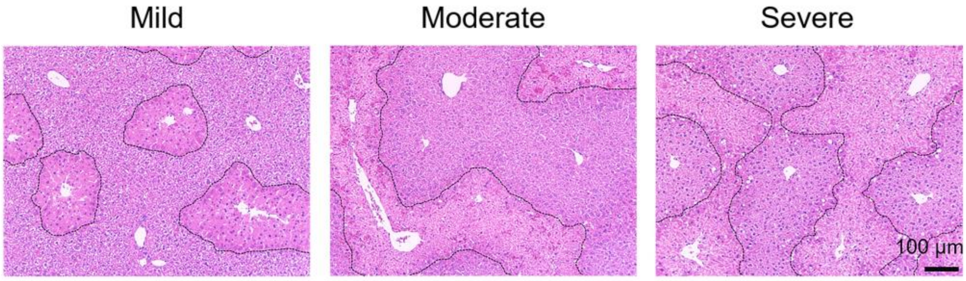

Figure S3. H&E staining in AILI with different grades. Original magnification, ×100; Scale bar, 100 µm. Mild:250 mg/kg; moderate, 450 mg/kg; severe, 550 mg/kg.

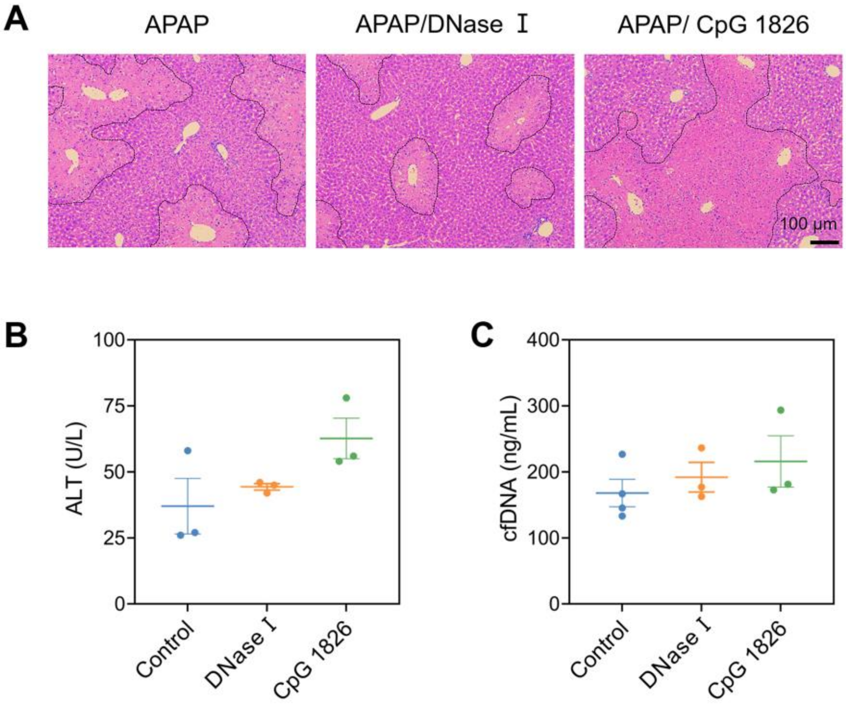

Figure S4. cfDNA amplifies liver damage in AILI. (A) H&E staining of AILI after DNase I or CpG ODN 1826 treatment. Original magnification, ×100; Scale bar, 100 μm. (B, C) Circulating ALT and cfDNA were determined after treatment of DNase I and CpG ODN 1826 in normal mice (n = 3-4 for group). Data are expressed as means ± SD, $^{*}P < 0.05$, $^{**}P < 0.01$, $^{***}P < 0.001$.

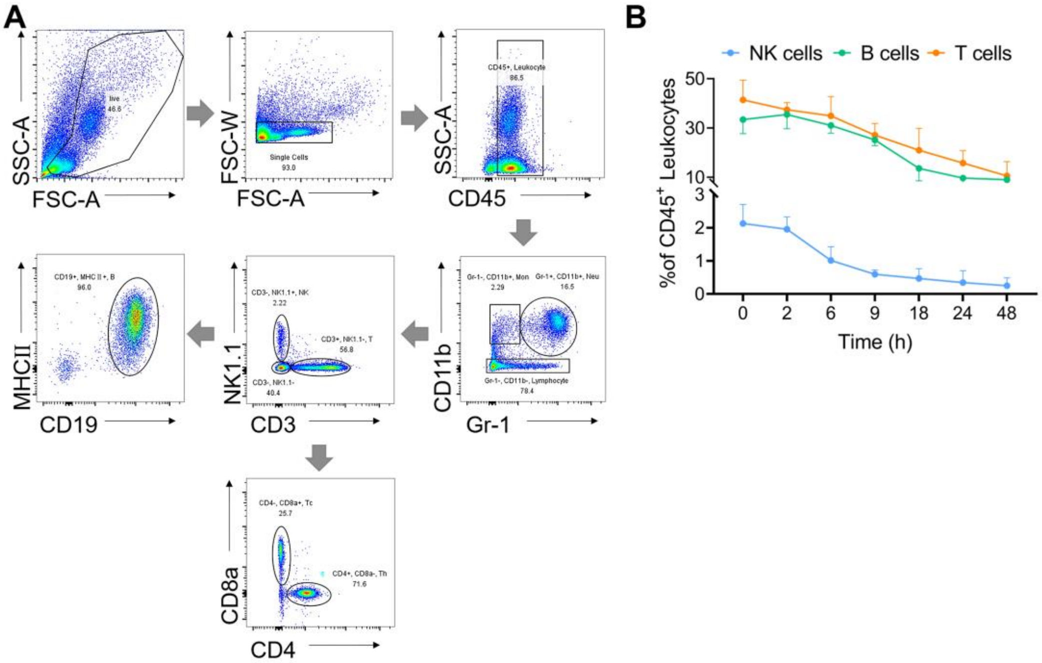

Figure S5. The transformation of leukocytes in peripheral blood of AILI (450 mg/kg). (A) Distinct leukocytes in peripheral blood were stained with indicated antibodies and assessed by flow cytometry at different time points after APAP. The gating strategy to identify neutrophils ($CD11b^+$ and $Gr\text{-}1^+$), monocytes ($CD11b^+$ and $Gr\text{-}1^-$), NK cells ($CD11b^-$ and $NK1.1^+$), B cells ($CD11b^-$, $CD19^+$ and MHC II^+), T cells ($CD11b^-$ and $CD3^+$) and its subtypes are shown. (B) The dynamics of NK cells, B cells and T cells (n = 3 for each time point). Data are expressed as means ± SD.

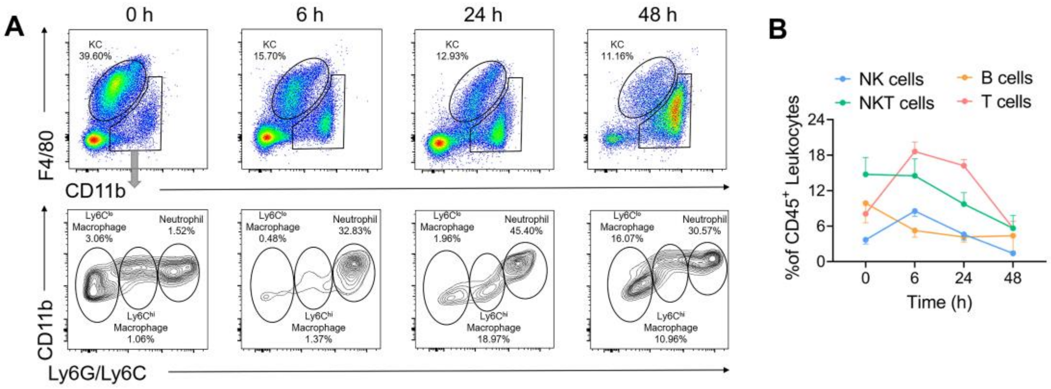

Figure S6. The transformation of leukocytes in liver of AILI (450 mg/kg). (A) Distinct myeloid cells in liver were stained with indicated antibodies and assessed by flow cytometry at different time points after APAP. The gating strategy to identify Kupffer cells (CD11b^{low} and F4/80^{high}), neutrophils (CD11b^{high} and Gr-1^{high}), Ly6C^{lo} macrophages (CD11b$^{high/intermediate}$ and Gr-1^{low}) and Ly6C^{hi} macrophages (CD11b$^{high/intermediate}$ and Gr-1^{intermediate}) and representative FACS plots are shown. (B) The dynamics of NK cells, NKT cells, B cells and T cells in liver (n = 3 for each time point). Data are expressed as means ± SD.

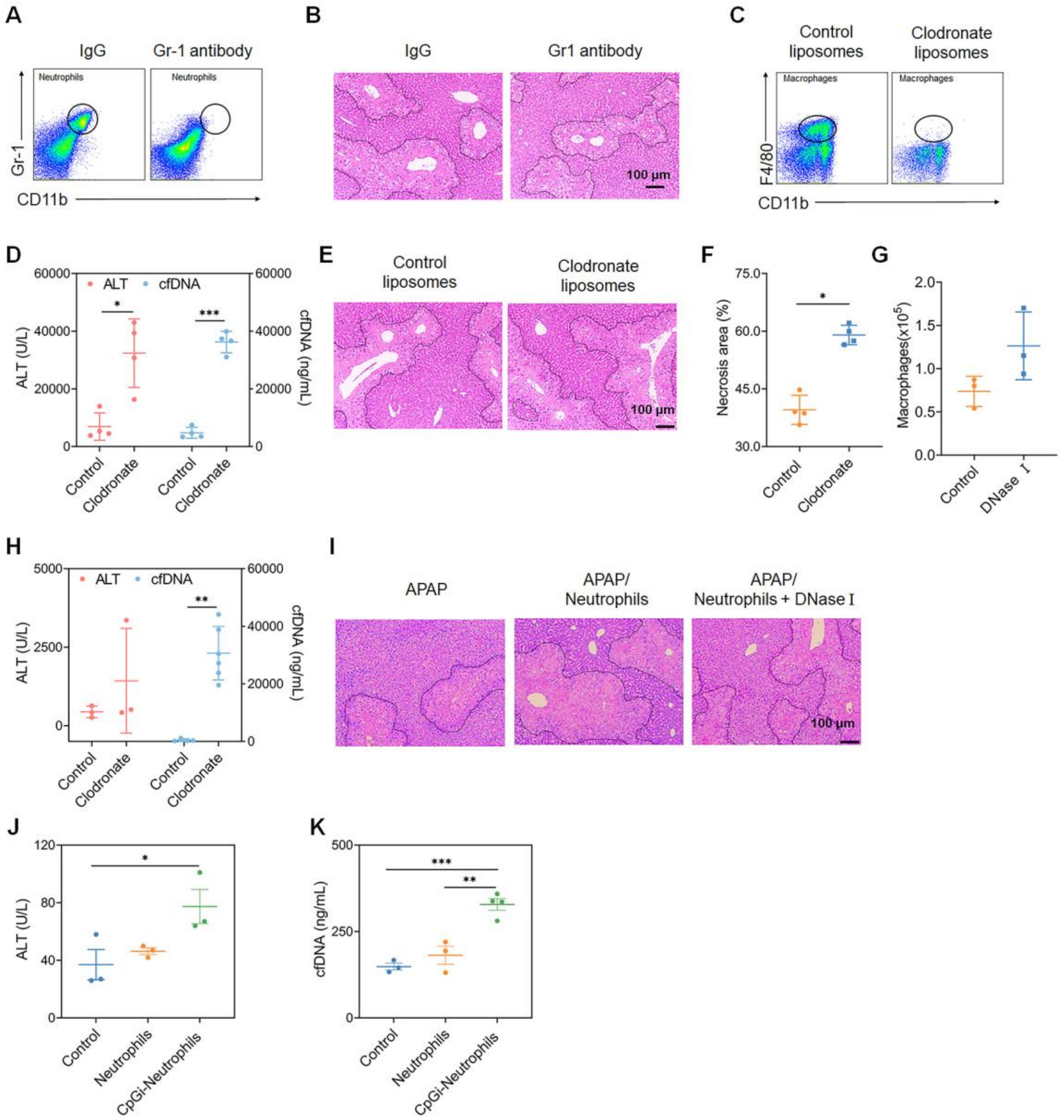

Figure S7. cfDNA amplifies liver damage and inflammation through neutrophils. (A) Anti-mouse Ly6G/Ly6C or IgG2b (100 μg/dose, i.p.) was administrated at 24 and 4 h before APAP (450 mg/kg) challenge to deplete neutrophils. (B) H&E staining. Original magnification, ×100; Scale bar, 100 μm. (C) Clodronate liposomes or control liposomes (250 μL, i.p.) were given at 48 h before APAP challenge to deplete macrophages. The gating strategy to identify neutrophils ($CD11b^{high}$ and $Gr\text{-}1^{high}$) and macrophages ($F4/80^{+}$) are shown. (D-F) Circulating ALT, cfDNA, H&E staining and necrosis area of liver after macrophages depletion at 24 h in AILI mice. (G) Variation of hepatic macrophages in AILI mice after DNase I treatment. (H) Circulating ALT and cfDNA levels after depletion of macrophages at 24 h in CpG-induced liver injury. The depletion of macrophages was realized as above described before CpG challenge. n = 3-6 for each group. (I) H&E staining after adoptive transfer experiment in AILI. Original magnification, ×100; Scale bar, 100 μm. (J, K) Circulating ALT and cfDNA after adoptive transfer experiment in normal mice (n = 3-5 for each group). Data are expressed as means ± SD, $^{*}P < 0.05$, $^{**}P < 0.01$, $^{***}P < 0.001$.

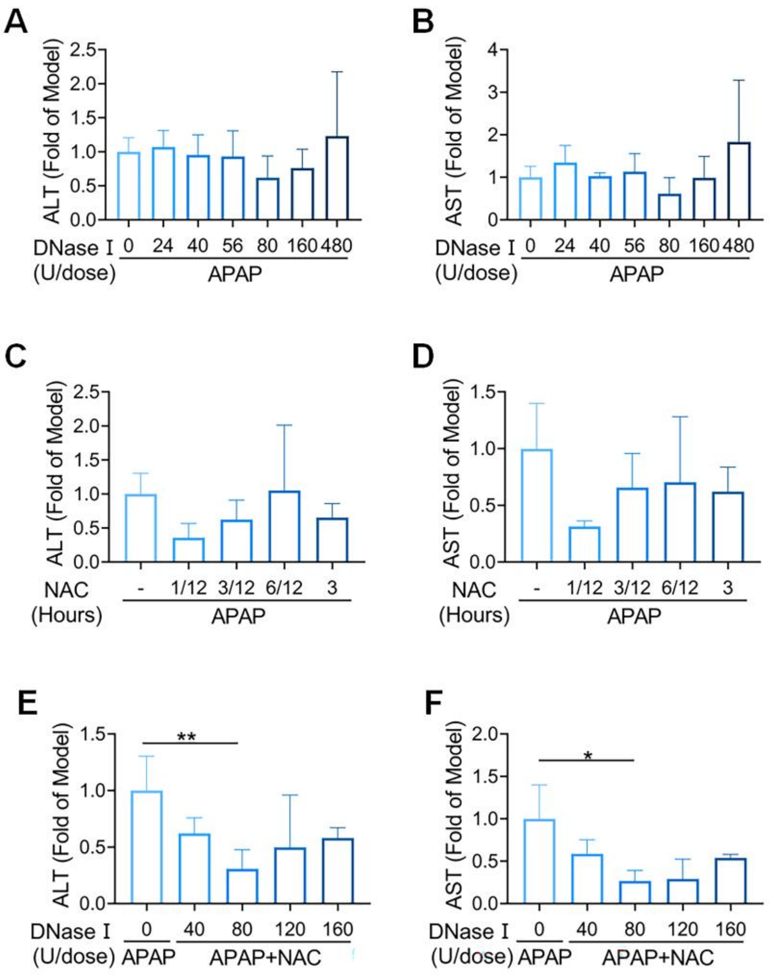

Figure S8. The optimization of drug administration *in vivo*. The levels of circulating ALT and AST after (A-B) DNase I treatment at different doses, (C-D) NAC treatment at different time points, (E-F) combination of NAC and different doses of DNase I (n = 3-7 for each group). Data are expressed as means ± SD, $^{*}P < 0.05$, $^{**}P < 0.01$, $^{***}P < 0.001$.

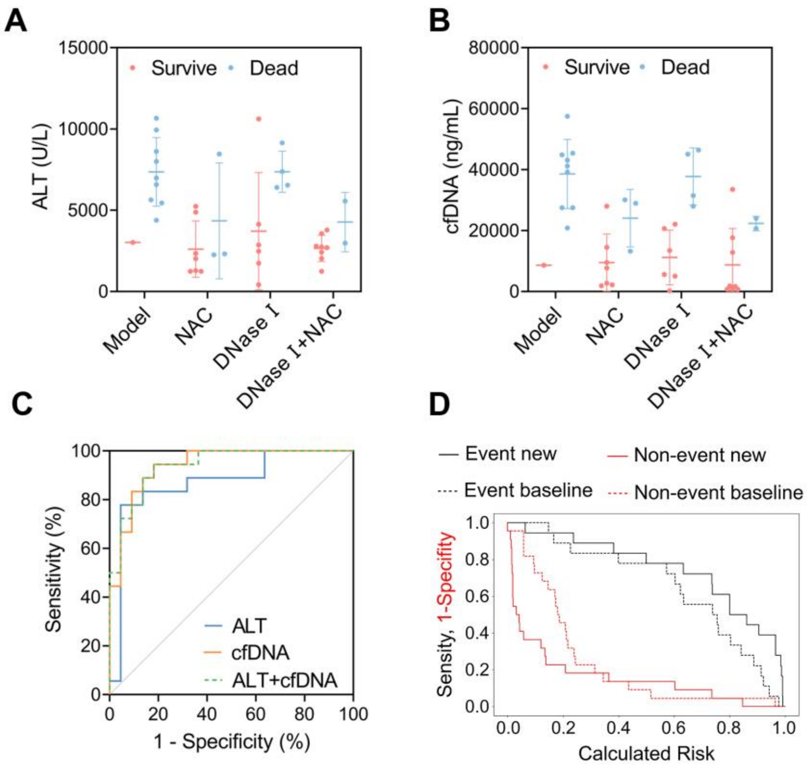

Figure S9. cfDNA is a valuable biomarker to predict the outcome and prognosis of AILI. (A, B) The levels of circulating ALT and cfDNA were measured at 12 h in dead or survival mice in severe AILI (550 mg/kg, n = 10 for each group). Data are expressed as means ± SD. (C) ROC curve analysis showed the potential of cfDNA, ALT and ALT + cfDNA to predict the outcome of AILI (550 mg/kg, n = 40). (D) Risk assessment of baseline model (ALT) and new model with the addition of cfDNA to predict outcome. Subjects who did not survive (event, black line) and survive (non-event, red line) are shown respectively (550 mg/kg, n = 40).

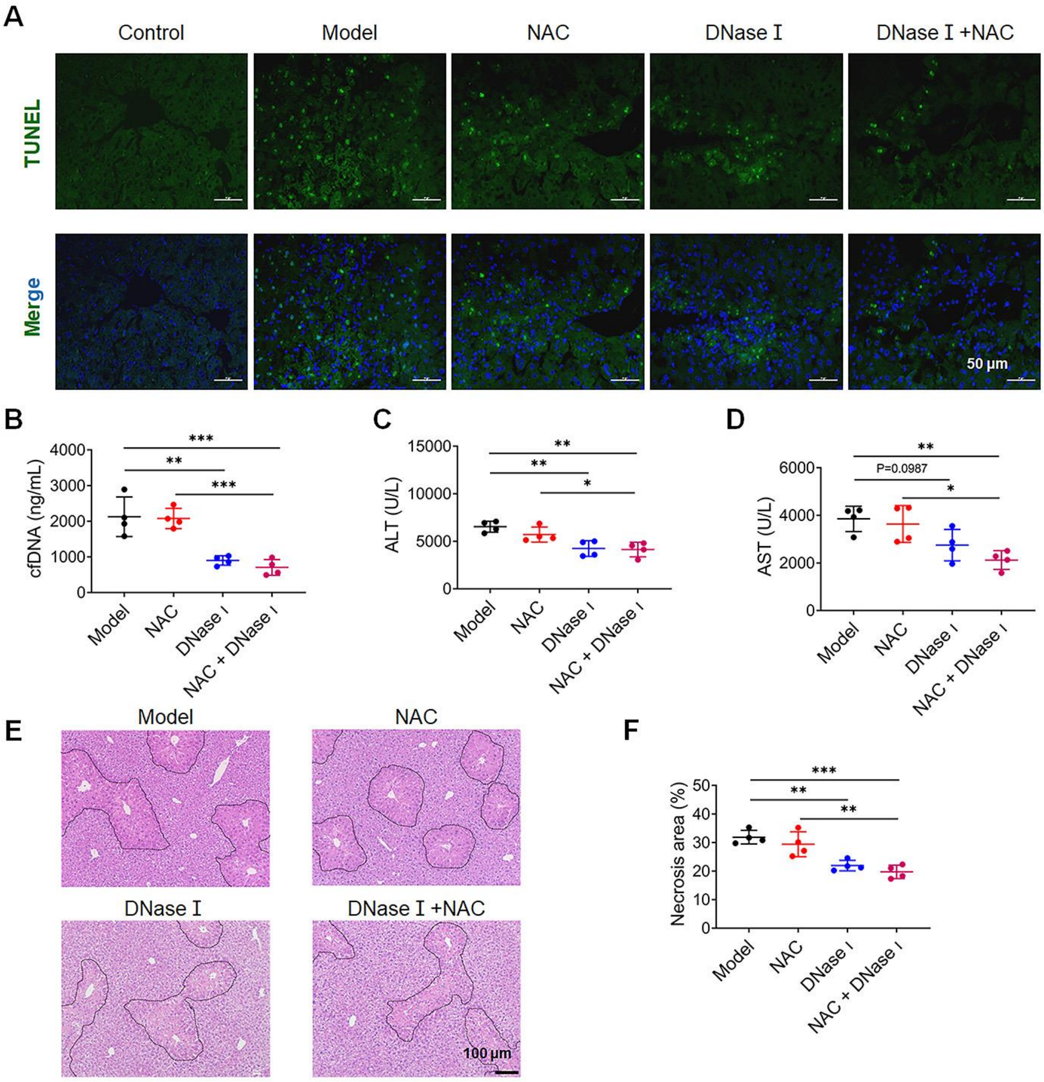

Figure S10. cfDNA is a therapeutic target in AILI. (A) TUNEL staining of the liver. Original magnification, ×200; Scale bar, 50 μm. (B-D) The levels of circulating cfDNA, ALT and AST at 24 h after APAP. (E) Representative H&E staining of the liver. Original magnification, ×100; Scale bar, 100 μm. (F) The levels of necrosis area at 24 h after APAP (250 mg/kg, n = 3-5 for each group). Data are expressed as means ± SD, *P < 0.05, **P < 0.01, ***P < 0.001.

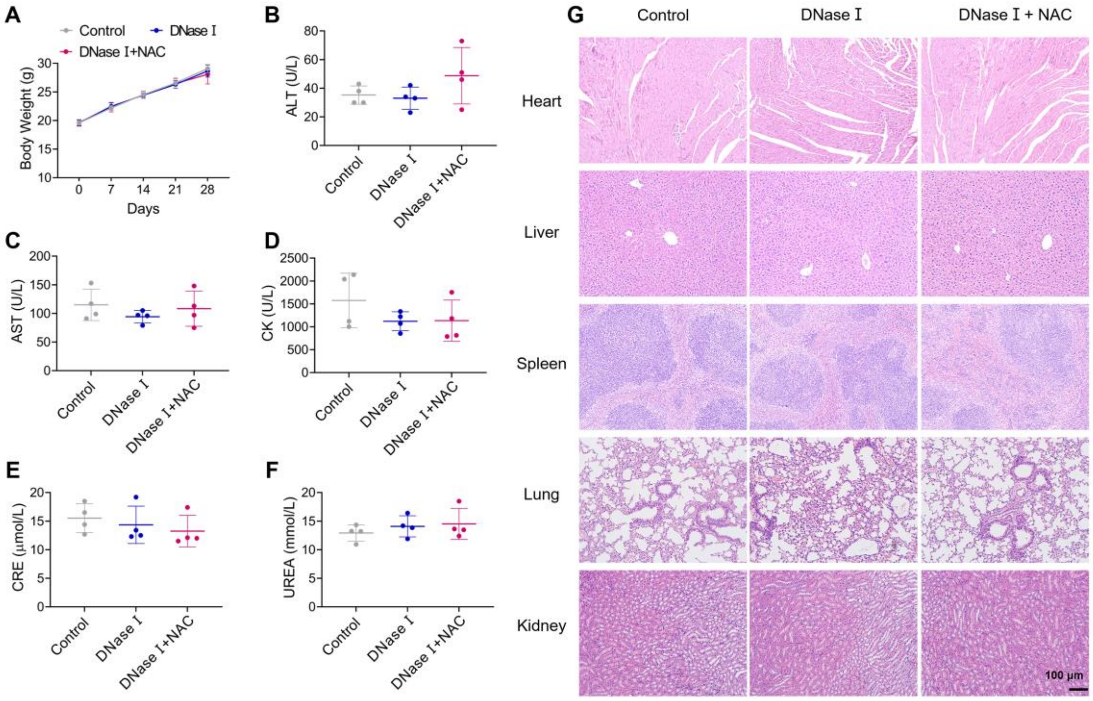

Figure S11. DNase I and NAC showed no obvious toxicity *in vivo*. Normal mice were treated with NAC (150 mg/kg, i.p.) and DNase I (80U/dose, i.v.) on day 1. (A) Body weight was monitored every week until 28 days. (B-F) The levels of circulating ALT, AST, CK, CRE and UREA were determined on day 28 (n = 4 for each group). Data are expressed as means ± SD, $^{*}P < 0.05$, $^{**}P < 0.01$, $^{***}P < 0.001$. (G) H&E staining of heart, liver, spleen, lung and kidney on day 28. Original magnification, ×100; Scale bar, 100 μm.

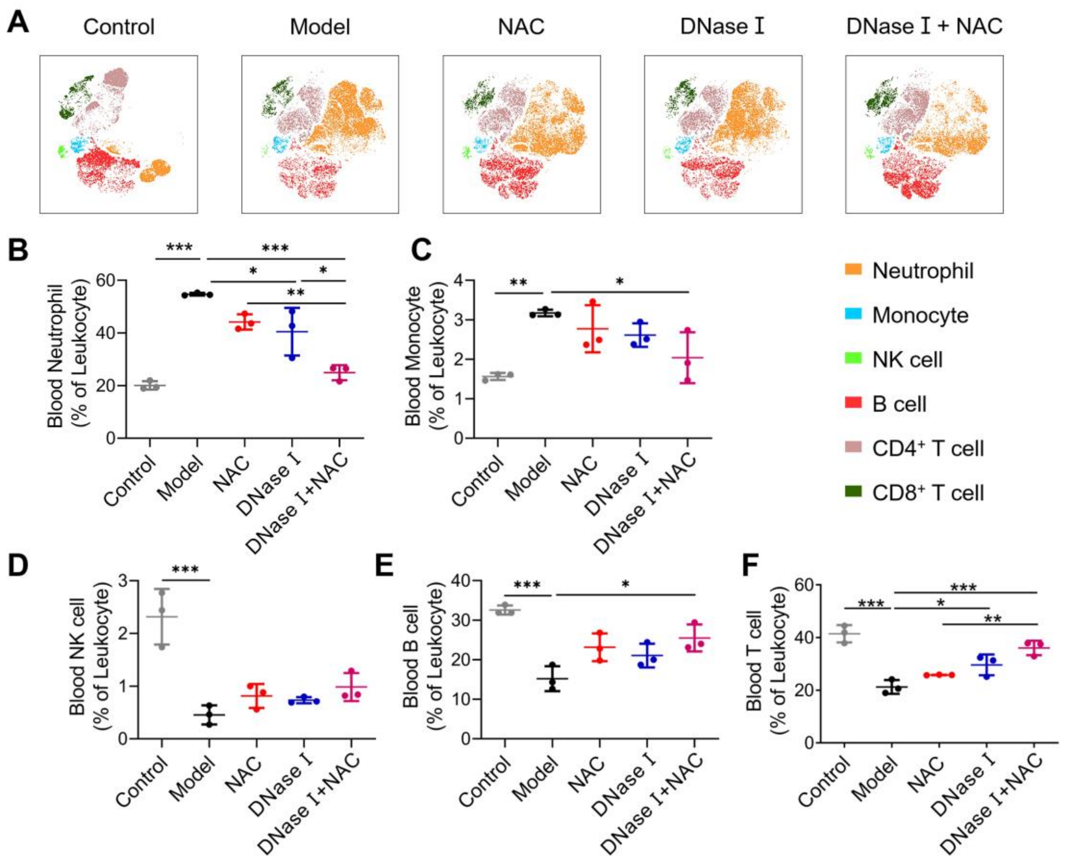

Figure S12. The variation of leukocytes in peripheral blood after treatment. (A) t-SNE analysis of leukocytes in peripheral blood at 24 h following APAP (250 mg/kg). (B-F) The proportion of neutrophils, monocytes, NK cells, B cells and T cells in peripheral blood at 24 h following APAP (n = 3 for each group). Data are expressed as means ± SD, $^{*}P < 0.05$, $^{**}P < 0.01$, $^{***}P < 0.001$.

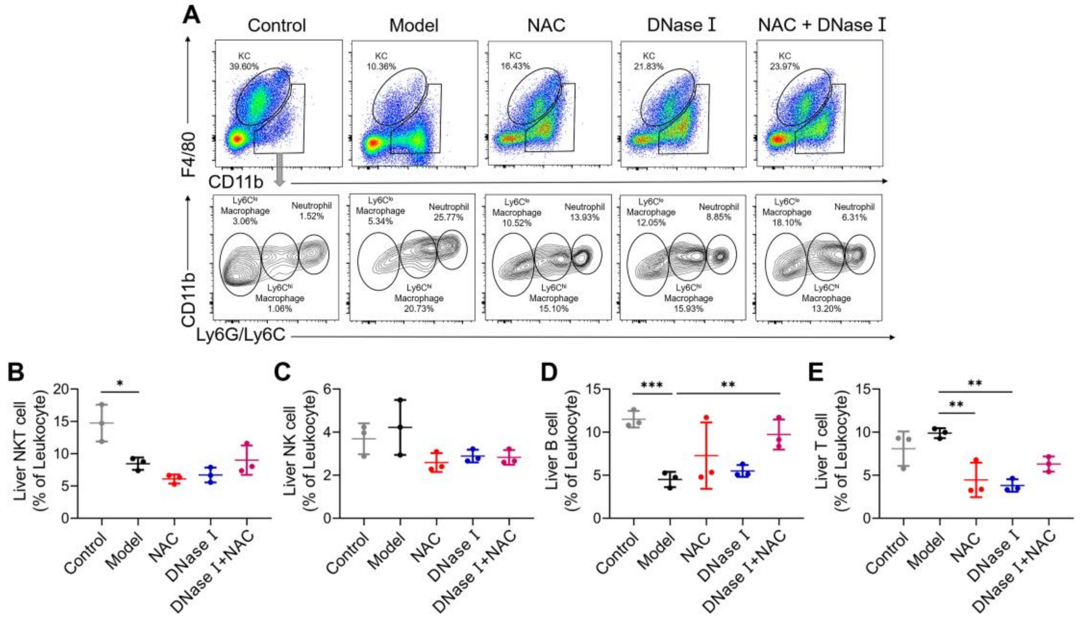

Figure S13. The variation of leukocytes in liver after treatment. (A) Distinct myeloid cells in liver were stained with indicated antibodies and assessed by flow cytometry at 24 h following APAP (250 mg/kg). The gating strategy to identify Kupffer cells ($CD11b^{low}$ and $F4/80^{high}$), neutrophils ($CD11b^{high}$ and $Gr\text{-}1^{high}$), $Ly6C^{lo}$ macrophages ($CD11b^{high/intermediate}$ and $Gr\text{-}1^{low}$) and $Ly6C^{hi}$ macrophages ($CD11b^{high/intermediate}$ and $Gr\text{-}1^{intermediate}$) and representative FACS plots are shown. (B-E) The proportion of NKT cells, NK cells, B cells and T cells in liver at 24 h (n = 3 for each group). Data are expressed as means ± SD, $^{*}P < 0.05$, $^{**}P < 0.01$, $^{***}P < 0.001$.

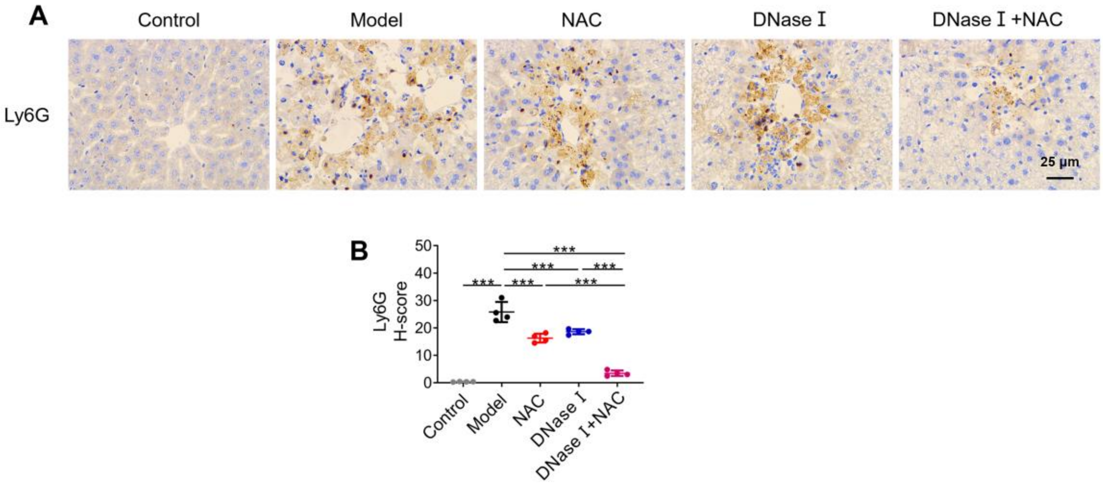

Figure S14. IHC of Ly6G in liver. (A) Immunohistochemical staining of liver for Ly6G. Original magnification, ×400; Scale bar, 25 μm. (B) Quantification of Ly6G expression in liver. (n = 4 for each group). Data are expressed as means ± SD, $^{*}P < 0.05$, $^{**}P < 0.01$, $^{***}P < 0.001$.

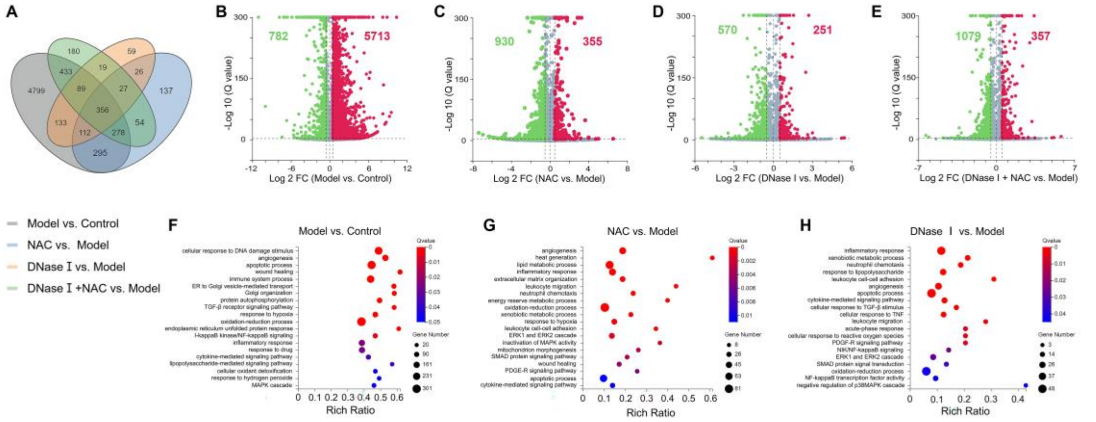

Figure S15. Analysis and GO functional enrichment of DEGs. (A) Venn diagram was performed based on DEGs (|log2 FC| ≥ 0.5 and Q < 0.001). Volcano plots were performed to show the identified upregulated and downregulated genes between (B) Model vs. Control, (C) NAC vs. Model, (D) DNase I vs. Model and (E) DNase I + NAC vs. Model. DEGs between (F) Model vs. Control, (G) NAC vs. Model, (H) DNase I vs. Model were analyzed by biological process of GO enrichment analysis. The 20 enriched biological processes are shown as bubble diagram.

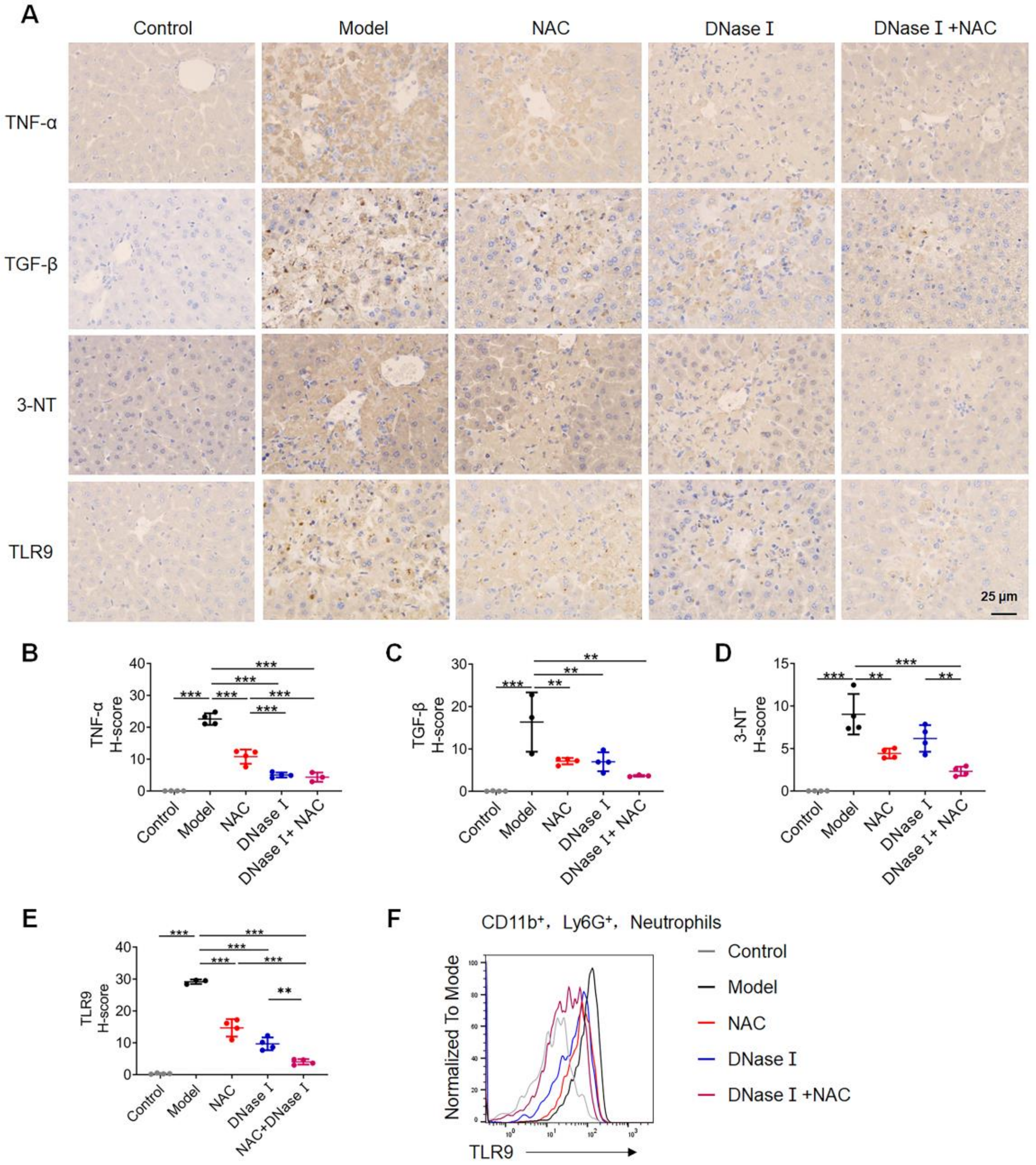

Figure S16. Combination therapy of DNase I and NAC reverses inflammation and oxidative stress in AILI. (A) Immunohistochemical staining of liver for TNF-α, TGF-β, 3-NT and TLR9 at 24 h following APAP. Original magnification, ×400; Scale bar, 25 μm. Quantification of (B) TNF-α, (C) TGF-β, (D) 3-NT and (E) TLR9 expression in liver. (F) TLR9 expression in hepatic neutrophils (n = 3-4 for each group). Data are means ± SD, $^{*}P < 0.05$, $^{**}P < 0.01$, $^{***}P < 0.001$.
